# Microbial Communities on Seafloor Basalts at Dorado Outcrop Reflect Level of Alteration and Highlight Global Lithic Clades

**DOI:** 10.3389/fmicb.2015.01470

**Published:** 2015-12-23

**Authors:** Michael D. Lee, Nathan G. Walworth, Jason B. Sylvan, Katrina J. Edwards, Beth N. Orcutt

**Affiliations:** ^1^Department of Biological Sciences, University of Southern CaliforniaLos Angeles, CA, USA; ^2^Department of Oceanography, Texas A&M UniversityCollege Station, TX, USA; ^3^Bigelow Laboratory for Ocean SciencesEast Boothbay, ME, USA

**Keywords:** basalt, geomicrobiology, oceanic crust, microbe–mineral interactions, biogeography

## Abstract

Areas of exposed basalt along mid-ocean ridges and at seafloor outcrops serve as conduits of fluid flux into and out of a subsurface ocean, and microbe–mineral interactions can influence alteration reactions at the rock–water interface. Located on the eastern flank of the East Pacific Rise, Dorado Outcrop is a site of low-temperature (<20°C) hydrothermal venting and represents a new end-member in the current survey of seafloor basalt biomes. Consistent with prior studies, a survey of 16S rRNA gene sequence diversity using universal primers targeting the V4 hypervariable region revealed much greater richness and diversity on the seafloor rocks than in surrounding seawater. Overall, Gamma-, Alpha-, and Deltaproteobacteria, and Thaumarchaeota dominated the sequenced communities, together making up over half of the observed diversity, though bacterial sequences were more abundant than archaeal in all samples. The most abundant bacterial reads were closely related to the obligate chemolithoautotrophic, sulfur-oxidizing *Thioprofundum lithotrophicum*, suggesting carbon and sulfur cycling as dominant metabolic pathways in this system. Representatives of Thaumarchaeota were detected in relatively high abundance on the basalts in comparison to bottom water, possibly indicating ammonia oxidation. In comparison to other sequence datasets from globally distributed seafloor basalts, this study reveals many overlapping and cosmopolitan phylogenetic groups and also suggests that substrate age correlates with community structure.

## Introduction

Areas of exposed oceanic lithosphere comprise a substantial proportion of the seafloor. The global mid-ocean ridge system generates new crust at an average rate of ~3.3 km^2^ year^−1^ (German and Von Damm, 2004). This contributes to ridge flanks having constantly-replenished, large stretches of unsedimented basalt totaling more than 10^6^ km^2^ worldwide (Edwards et al., 2005). Dwarfing this, however, are the estimated millions of seamounts and outcrops >100 m in relief that extend above sedimentation lines (Wessel et al., 2010). Conservative estimates including only those taller than 1 km pin this area at nearly 30 × 10^6^ km^2^ worldwide, providing a potential biome larger than the global continental shelf (Etnoyer et al., 2010). These regions also serve as conduits of fluid flux into and out of a subsurface ocean (Edwards et al., 2005), facilitating ongoing exchanges between the crust and seawater that contribute as much chemically-altered fluid to the ocean as the totality of global riverine inputs (Elderfield and Schultz, 1996; Wheat and Mottl, 2000).

At the rock–water interface, chemically-reduced substrates hosted by basalts meet an oxidative ocean, supporting spontaneous oxidation–reduction (redox) reactions. These reactions cause slow weathering of basalts at low temperatures, granting microorganisms the opportunity to manipulate this energy transfer, accelerating redox processes by unknown magnitudes and forming a chemolithotrophic foundation for basalt-hosted biomes. Accounting for Fe and S oxidation alone, the most dominant reduced substrates found in basalt, it has been estimated that this trophic foundation may extend 500 m down into the crust and can theoretically support the production of as much as 10^11^ g of fixed C per year (Bach and Edwards, 2003; Orcutt et al., 2015) In addition to Fe and S, basaltic crust hosts reduced Mn as well as P, Ni, and other trace elements integral to microbial processes (Staudigel et al., 2008). Driven by redox reactions, as fresh seafloor basalts weather, an outer rind develops that is primarily composed of Mn and Fe (>30%) and other elements sourced from the basalt itself; however, similar to ferromanganese nodules found throughout the seafloor, these outer rinds also serve as nuclei that continue to accrete additional biologically significant metals including Co, Cu, Mo, Zn, Pb, and others as the basalt ages (Mero, 1962). Some have argued this fluid-delivery mechanism is actually the primary source of support for basalt-hosted microbial communities (Templeton et al., 2009).

Although ocean crust comprises a significant fraction of the seafloor, the microbial communities resident within and on these rocks—and likely involved in the rock alteration processes described above—are poorly understood. Culturing efforts have yielded otherwise unknown Mn oxidizers (Templeton et al., 2005) and Fe oxidizers (Edwards et al., 2003, 2004; Rogers et al., 2003; Daughney et al., 2004), suggesting that these microorganisms may play a role in the weathering of rock at the seafloor. Culture-independent molecular techniques aimed at phylogeny have also been successful in probing the microbial communities of seafloor basalts. Sequences from the Knipovich Ridge in the Atlantic Arctic have revealed Gamma-, Alpha-, Delta-, and Epsilonproteobacteria, as well as Actinobacteria, Chloroflexi, Firmicutes, and Bacteroidetes within Bacteria, and Marine Group I Crenarchaeota (now known as Thaumarchaeota; Brochier-Armanet et al., 2008) within the Archaea (Thorseth et al., 2001; Lysnes et al., 2004). Samples from the Juan de Fuca Ridge (JdF) were found to contain Gamma-, Beta-, and Epsilonproteobacteria (Rogers et al., 2003). In addition to these previously mentioned taxa, two other studies with samples from the East Pacific Rise (EPR), Loihi Seamount, and JdF also recovered Gemmatimonadetes, Nitrospirae, Planctomycetes, and Verrucomicrobia (Santelli et al., 2008; Mason et al., 2009). Larger clone libraries revealed basalts host extremely diverse microbial communities and suggested that rock alteration positively correlated with richness and diversity (Santelli et al., 2009). A study looking at very young, newly erupted samples (<10 years) from Vailulu'u Seamount in the southwest Pacific identified Alpha-, Beta-, Delta-, Epsilonproteobacteria, and Bacteroidetes (Sudek et al., 2009), while a survey of a relatively much older site, Takuyo Seamount (~80 mya) near Japan, detected all of the taxa mentioned above and additionally Caldithrix, Chlamydiae, and clones belonging to the groups BRC1, KSB, NKB19, OP11, OP3, SAR406, and SBR1093, and suggested that aged basalts with developed Mn crusts are as or more diverse than younger basalts (Nitahara et al., 2011). Generally consistent findings of these prior phylogenetic surveys include that these substrates host some of the most diverse communities known, are more abundantly characterized by bacteria over archaea [though quantitative Polymerase Chain Reaction (qPCR) has yielded at least one exception to this; Nitahara et al., 2011], and are consistently dominated by Gamma- and Alphaproteobacteria. The broad similarities detected in communities over large geographic stretches has led some to suggest there are conserved major taxa that are ubiquitous with regard to seafloor basalts (Mason et al., 2007, 2009; Santelli et al., 2008).

Previous studies of microbial diversity hosted on seafloor basalts have focused on either relatively young (<~3 mya; Thorseth et al., 2001; Rogers et al., 2003; Lysnes et al., 2004; Santelli et al., 2008; Mason et al., 2009; Sudek et al., 2009) or old (~80 mya; Nitahara et al., 2011) ocean crust, precluding an assessment of how microbial communities may develop with age of the seafloor. Moreover, prior work has focused on basalts from actively venting high-temperature (>200°C) hydrothermal environments (Rogers et al., 2003) or quiescent seafloor habitats (Thorseth et al., 2001; Lysnes et al., 2004; Santelli et al., 2008; Mason et al., 2009; Sudek et al., 2009; Nitahara et al., 2011), but nothing is known about microbial community structure on basalts from low-temperature (<25°C) venting environments postulated to be abundant seafloor features (Elderfield and Schultz, 1996; Wheat and Mottl, 2000; Wessel et al., 2010).

**Figure 1:**
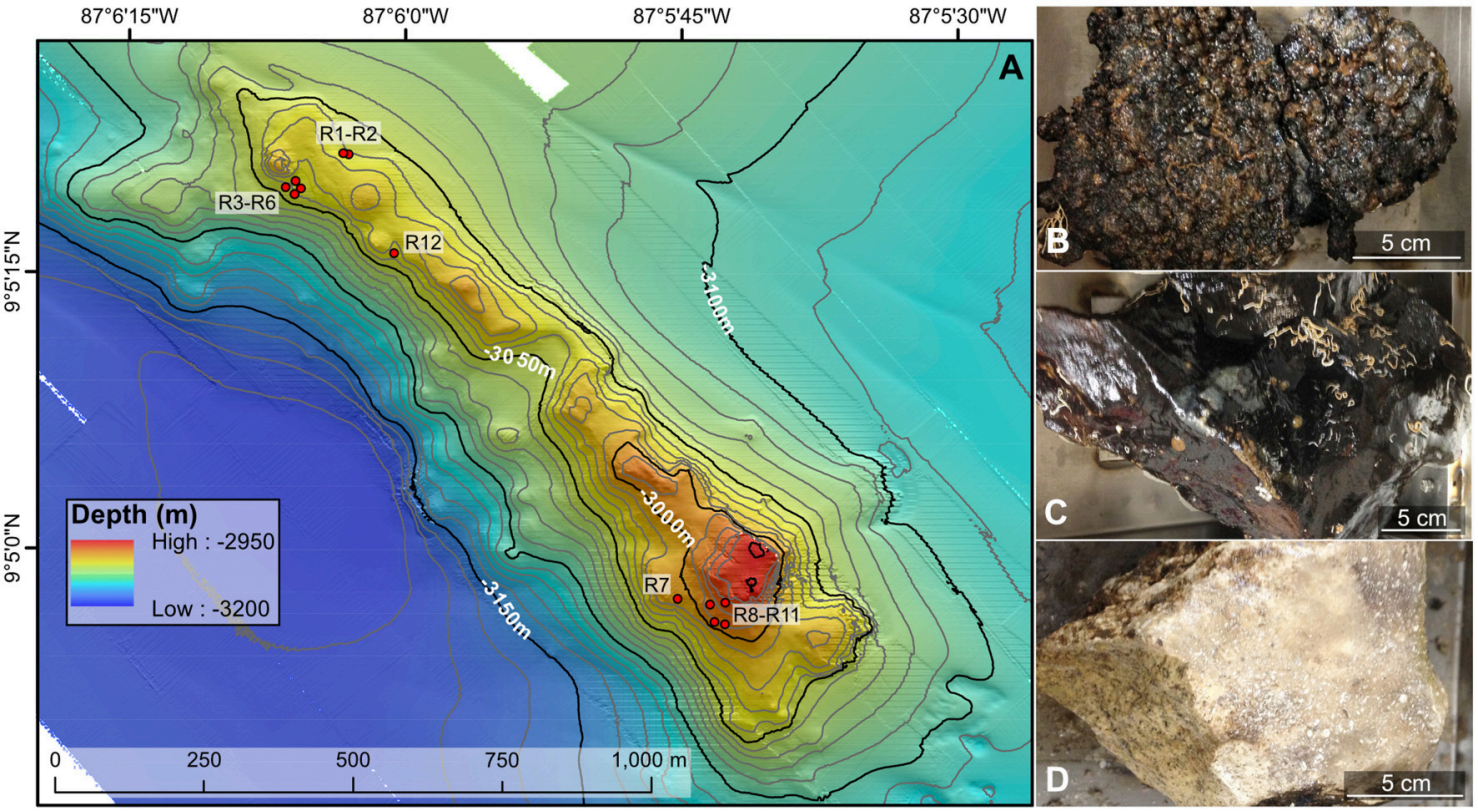
**Bathymetric map (A) and example rock types (B–D) collected from the Dorado Outcrop offshore Costa Rica in the Pacific Ocean**. **(A)** Locations of the 12 rock samples examined in this study are indicated with red circles. The underlying north–south oriented map was created shipboard by Samuel Hulme and Chris Treboal from bathymetric data collected by the AUV *Sentry* at heights of 5, 30, and 60 m above bottom, with 10 m water depth contours (data are publicly available through the National Oceanic and Atmospheric Administration's National Centers for Environmental Information database). **(B–D)** Examples of highly oxidized, manganese encrusted basalt **(B)**, less altered basalt **(C)**, and lithified carbonate **(D)** rock types examined in this study, with scale bars to indicate size. Photographs by Michael Lee.

Located 200 km west of Costa Rica on the eastern flank of the East Pacific Rise, Dorado Outcrop (Figure 1A) is an environment that allows assessment of both of these conditions as it is comprised of 23 Ma seafloor (Wheat and Fisher, 2008) where cool (10–20°C) hydrothermal fluids were predicted to be venting after transiting from nearby Tengosed Seamount located ~20 km away (Hutnak et al., 2008; Wheat and Fisher, 2008). Sediment porewater chemistry from on and around Dorado Outcrop indicates minimally altered fluids exiting the seafloor (i.e., nitrate is present at 42.3 μmol/kg in bottom water and estimated at 40.9 μmol/kg in venting fluids; Wheat and Fisher, 2008). This is in contrast to mid-ocean ridge and hotspot volcanoes, where the chemical composition of diffuse flow vent fluids is very different from background seawater. Here, we analyze 16S rRNA gene sequences (focusing on the V4 hypervariable region) from basalts collected from Dorado Outcrop to provide the first survey of microbial communities inhabiting basalts recovered from a low-temperature venting, “middle-aged” outcrop. Building upon previous work investigating the biogeography of seafloor-exposed basalts, we further elucidate globally distributed monophyletic clades, supporting the observation that the composition of deep-sea mineral substrates plays a larger role than other factors (e.g., geography, temperature, depth) in determining mineral-attached microbial community structure (Santelli et al., 2008; Mason et al., 2009; Toner et al., 2013) and further support the notion of a crust-associated clade within the Thaumarchaeota as has been put forward previously (Mason et al., 2007).

## Materials and methods

### Sample collection

Dorado Outcrop (Figure 1A) is located 200 km west of Costa Rica on the Cocos Plate (Wheat and Fisher, 2008). Its surface is ~3000–3100 m below the sea surface and rises to a height of ~80 m above the surrounding seafloor (Wheat and Fisher, 2008). Over the span of December 7–23, 2013, 12 seafloor rock samples (11 basalts and 1 lithified carbonate, hereafter R1–R12; Table 1, Figure S1) were collected from across Dorado Outcrop while aboard *R/V Atlantis* during cruise AT26-09 following previously developed protocols (Santelli et al., 2008). Immediately prior to collection, the temperature of fluids venting from cracks in the outcrop was measured using the “high temperature” thermocouple available from the *ROV Jason II*. Using the *ROV*, all samples were placed in plastic “bioboxes” that were sealed at depth to maintain them in seawater from where they were collected. Once onboard the ship, samples were immediately photographed and processed in a flame-sterilized steel box with a flame-sterilized hammer and chisel. Most samples possessed visibly altered outer rinds that were chiseled off for molecular analysis. For those lacking an altered rind (namely the lithified carbonate sample), the exterior was targeted to chip off small fragments of rock. Samples for DNA analysis were placed in either sterile whirl-pak bags or centrifuge tubes and frozen at −80°C. Locations of the collection sites are marked on Figure 1A and coordinates can be found in Table 1. In addition to the basalts, two bottom water samples were collected using a Niskin bottle mounted to an elevator, and 1.25 L were filtered onto 0.2 μm pore size polycarbonate Nucleopore filters that were then frozen at −80°C.

**Table 1:** **Characteristics of the rock and background seawater samples investigated in this study including sample description, ROV ***Jason-II*** dive number, Dorado Outcrop site marker classification, latitude, longitude, water depth, and water temperature**.

**ID**	**Description**	**Dive**	**Site**	**Lat. (N)**	**Lon. (W)**	**Water depth (m)**	**Water temp^a^ (°C)**
R1	Altered basalt, >1 cm thick black Mn rind	J2-752	14	9.089	87.101	3039	8.6
R1A^*^	Replicate extraction of above	J2-752	14	9.089	87.101	3039	8.6
R2	Altered basalt, >1 cm thick black Mn rind	J2-752	14	9.089	87.101	3039	8.6
R3	Altered basalt, >1.5 cm thick black Mn rind	J2-752	A	9.089	87.102	3041	12.7
R4	Altered basalt, >1 cm thick black Mn rind	J2-752	A	9.089	87.102	3041	12.7
R5	Altered basalt, >1.5 cm thick black Mn rind	J2-752	A	9.089	87.102	3041	12.7
R6	Altered basalt, >1 cm thick black Mn rind	J2-752	A	9.089	87.102	3041	12.7
R7	Lithified carbonate	J2-757	M	9.099	87.096	3011	n.d.
R8	Glassy basalt, 1 cm thick black and orange rind	J2-757	D	9.082	87.095	3007	13.5
R9	Glassy basalt, 1 cm thick black Mn rind	J2-757	K	9.082	87.095	3007	13.7
R10	Glassy basalt, thin black and orange rind	J2-757	K	9.082	87.095	3007	13.7
R11	Glassy basalt, thin black and orange rind, green veneer	J2-757	F	9.082	87.095	3007	7.3
R12	Basalt, thin black rind	J2-757	B	9.088	87.101	3024	n.d.
R11-BF	Green veneer from R-11	J2-757	F	9.082	87.095	3007	7.3
BW-1	1.25 L Bottom seawater (elevator Niskin)	n.a.	n.a.	9.083	87.103	3183	2.0
BW-2	1.25 L Bottom seawater (elevator Niskin)	n.a.	n.a.	9.081	87.101	3176	2.0

*Sample marked with asterisk indicates a technical replicate of sample R1—a separate part of the outer rind was analyzed. n.a., not applicable; n.d., not determined*.

a *Measurements were made with an uncalibrated thermocoupler (Jason II), and bottom water temperature was ~2°C*.

### DNA extraction and sequencing of the 16S rRNA gene

Frozen rock pieces were crushed in a flame-sterilized impact mortar into sand-sized grains which were then transferred to sterile plastic centrifuge tubes and stored at −80°C until DNA extractions were performed. DNA extractions were carried out with the FastDNA Spin Kit for Soil (MP Biomedicals, Santa Ana, CA, USA) following the manufacturer's specifications. About 0.5 g of crushed material were placed directly into the lysis tubes of the kit, as were the bottom water filters. Protocol blanks were performed with each extraction (no samples or DNA added to lysis tubes) to track the potential for contamination. DNA concentrations were quantified with the Qubit HS dsDNA Assay kit with a Qubit 2.0 Fluorometer (Life Technologies, Carlsbad, CA, USA) according to manufacturer protocols.

DNA extracts from the 12 rock samples (plus one technical replicate from the same sample), one green-colored, potential biofilm sample from R11 (Figure S2), two bottom water samples, and four protocol blanks for a total of 20 samples (Table 2) were sent for DNA sequencing by a commercial vendor (Molecular Research LP; MR DNA; Shallowater, TX, USA). Illumina MiSeq paired-end (2 × 300 base pair) tag sequencing was carried out using the Earth Microbiome Project universal primers 515f (5′-GTG CCA GCM GCC GCG GTA A-3′) and 806r (5′-GGA CTA CHV GGG TWT CTA AT-3′; Caporaso et al., 2012), which flank the V4 region of the 16S rRNA gene. Library preparation and sequencing was carried out at the facility. In brief, the 515f/806r PCR primers with 8-base barcodes on the forward primer were used in a PCR reaction with the HotStarTaq Plus Master Mix Kit (QIAGEN:USA, Valencia, CA, USA) under the following conditions: 94°C for 3 min, followed by 28 cycles of 94°C for 30 s, 53°C for 40 s, and 72°C for 1 min, and a final elongation step at 72°C for 5 min. PCR products were then run on a 2% agarose gel to check amplification and relative intensity of bands. Based on their DNA concentrations and molecular weight, multiple samples were pooled together in equal proportions, purified with Ampure XP beads, and then used to prepare the library by following the Illumina TruSeq DNA library preparation protocol.

**Table 2:** **DNA extraction and 16S rRNA gene sequencing characteristics of the rock and background seawater samples investigated in this study, including mass or volume of sample extracted for DNA, DNA concentration (in ng DNA g^**−1**^ or ng DNA ml^**−1**^), number of 16S rRNA gene sequence reads before and after filtering and removal of sequences from the protocol blanks, and number of predicted operational taxonomic units (OTUs) defined at the 97% or greater sequence similarity level before and after filtering and blank removal**.

**ID**	**Sample extracted (g or ml)**	**ng DNA g^−1^ or ng ml^−1^**	**Number of QC reads**	**Number of OTUs**	**Number of reads after filtering**	**Number of OTUs after filtering**
R1	0.38	572	155,483	10,893	135,466	1376
R1A^*^	0.40	343	207,831	13,329	178,760	1433
R2	0.45	105	239,591	11,203	210,764	1464
R3	0.39	212	225,789	13,111	195,939	1464
R4	0.43	5.9	243,465	9123	215,990	1249
R5	0.42	404	232,055	14,432	198,281	1517
R6	0.77	438	194,066	11,023	166,608	1412
R7	0.59	3.8	110,382	4272	93,015	1069
R8	0.57	14	166,861	5933	150,118	1190
R9	0.50	91	118,960	5647	109,163	1205
R10	0.55	196	159,781	4956	146,733	1141
R11	0.50	36	115,105	6297	103,064	1263
R12	0.43	422	197,018	16,553	164,991	1460
R11-BF	n.d.	n.d.	121,025	4103	110,244	810
BW-1	1250	b.d.l.	32,354	1031	21,218	381
BW-2	1250	b.d.l.	84,314	2328	70,775	577
Blank-1	n.a.	b.d.l.	24,651	425	n.a.	n.a.
Blank-2	n.a.	b.d.l.	9354	173	n.a.	n.a.
Blank-3	n.a.	b.d.l.	8008	159	n.a.	n.a.
Blank-4	n.a.	b.d.l.	7823	227	n.a.	n.a.

*Sample marked with asterisk indicates a technical replicate of sample R1. b.d.l., below detection limit; BF, biofilm; BW, bottom water; n.a., not applicable; n.d., not determined; OTU, Operational Taxonomic Unit; QC, quality controlled*.

In addition to MiSeq tag sequencing, one near-full-length 16S rRNA clone library was prepared from sample R5. Following extraction as noted above, DNA was amplified with GoTaq Green Master Mix (Promega, Madison, WI, USA) using universal bacterial primers B27f (5′-AGA GTT TGA TCM TGG CTC AG-3′) and U1492r (5′-GGT TAC CTT GTT ACG ACT T-3′; Lane, 1991). The PCR was performed as follows: 95°C for 5 min, then 30 cycles of 95°C for 30 s, 55°C for 30 s, and 72°C for 90 s, followed by a final 72°C for 10 min. After amplification, PCR product was purified with the QiaQuick PCR Purification Kit (QIAGEN:USA, Valencia, CA, USA), and cloned into the pCR 4 TOPO vector using the TOPO TA Cloning Kit (Invitrogen, Grand Island, NY, USA). Transformants were plated on LB agar containing 100 μg mL^−1^ ampicillin as per the manufacturer's instructions and incubated overnight. Ninety-six colonies were then selected and grown overnight in liquid culture. These were sent for Sanger sequencing at Beckman Coulter Genomics in Danvers, MA, USA and resulted in the recovery of 67 near full-length 16S rRNA gene sequences.

### Sequence data analysis

Tag data curation and processing were carried out using *mothur* v.1.34.4 (Schloss et al., 2009) following the *mothur* Illumina MiSeq Standard Operating Procedure (Kozich et al., 2013). Briefly, paired reads were joined into contigs and any sequences with ambiguous base calls or homopolymers longer than 8 bp were removed. These merged contigs were aligned to the mothur-recreated Silva SEED database from release v119 (Yarza et al., 2008). Sequences were then pre-clustered at a near 1% dissimilarity by ranking the sequences by abundance and merging the most rare with the most abundant using the *pre.cluster* command with diffs = 2, as this step has been shown to mitigate the generation of spurious sequences (Kozich et al., 2013). Chimeras were screened with UCHIME using *de novo* mode (Edgar et al., 2011) and removed from further processing and analysis. Sequences were clustered into Operational Taxonomic Units (OTUs) at 3% or less sequence dissimilarity using the average neighbor method. These are referred to throughout as OtuXXXXXX.

#### Treatment of extraction blanks and OTU filtering

OTUs recovered from the four protocol blank samples (which may reflect over-amplification of contaminant DNA from the DNA extraction or sequencing kit reagents, as has been observed previously (Champlot et al., 2010), or cross-contamination from actual samples during sample handling) were statistically treated to determine if the sequences should be removed from the actual sample datasets, based on the proportional abundance of the reads. For example, OTUs with only a few reads in the blank samples but a higher abundance of reads in the actual samples were not removed from the dataset, as they likely reflected “real” sequences that resulted in the blanks from cross-contamination. To be rigorous in the application of this cutoff criterion, the OTUs from the four blanks were combined and compared against all of the samples combined together and normalized (divided by four to account for the difference in sample numbers). If any OTU was represented by at least 50 sequences in the sample dataset and had at least an order of magnitude or more reads than in the blank dataset, it was retained. Otherwise, the OTU was removed from the dataset, as it was likely a result of kit contaminant amplification. Following removal of blank sequences, the dataset was filtered using a conservative minimum OTU abundance cutoff threshold of 0.005% of total reads, as has been recommended when a mock community is not incorporated with sequencing in order to further mitigate the generation of spurious OTUs (Bokulich et al., 2013). This precludes, however, performing any richness estimator calculations such as the Chao1 abundance estimator or the abundance-based coverage estimator (ACE), as these utilize singletons in their calculations.

#### OTU matrix visualizations and statistics

Visualizations, tests of significance, analysis of similarities (ANOSIM), and permutational analysis of variance (ANOVA) tests of the final OTU abundance matrix were carried out in *RStudio* version 0.98.1091 (Racine, 2012) using the package *vegan* version 2.3-0 (Oksanen, 2015) with default settings unless otherwise noted. Hierarchical clustering analyses were performed with Bray-Curtis, Jaccard, and Yue-Clayton dissimilarity indices. Non-metric multidimensional scaling (NMDS) ordinations were carried out using the function *metaMDS*(). Redundancy analysis (RDA), ANOSIM, and permutational ANOVA were performed to test for any correlations between community composition and sample collection location, groupings of basalts by level of alteration, or observed venting temperature at time of collection using the functions *rda*(), *anosim*(), and *adonis*() with 10,000 permutations. In the case of the RDA ordination, because ecological data containing very high abundances as well as many rare abundances can be misleading when calculating Euclidean distances on raw values, the data were first Hellinger transformed as this has been shown to be effective in ameliorating this problem (Legendre and Gallagher, 2001).

#### Clone library processing

For the clone library, near full-length contigs were assembled using *Geneious* v6.1.8 (Kearse et al., 2012). Sequences were oriented and trimmed manually and then screened for chimeras using the online program *Decipher* version 1.14.4 (Wright et al., 2012). The resulting sequences were used in phylogenetic tree construction and submitted to BLAST (Altschul et al., 1990) to search for nearest cultured neighbors as well as environmental samples.

#### Incorporation of other seafloor basalt studies

Multiple studies of seafloor basalts that contained clone sequence data for the 16S rRNA gene were examined in context with the MiSeq tag data generated by this study in an effort to delineate any globally distributed clades. This effort follows up on previous work (Mason et al., 2007) with data now available from more geographically disparate sites. The sites incorporated here include: a recently erupted sample (2004) from the Vailulu'u Seamount in the Southern Pacific Ocean, near American Samoa (Sudek et al., 2009); a sample from the East Pacific Rise from a recent eruption (1991), designated as 9N in this study (Mason et al., 2009); samples from the Loihi Seamount and South Point near the big island of Hawaii (labeled collectively as Loihi herein) that are <1000 years old (Santelli et al., 2008); older samples from the East Pacific Rise at <18,000 years labeled here as EPR (Santelli et al., 2008); samples from the Juan de Fuca Ridge (JdF) in the Northern Pacific that are 100 kya and 3.3 mya (Mason et al., 2009); Mn crusts from the 80 mya Takuyo-Daigo Seamount in the Northwest Pacific (Nitahara et al., 2011); basalts from Lau Basin (Sylvan et al., 2013); and basalts from an Arctic spreading ridge in the northern Atlantic (Thorseth et al., 2001; Lysnes et al., 2004). Sequences were gathered from GenBank and used for identifying overlapping OTUs and generating phylogenetic trees. Previous studies were selected for comparison only if they had sequence information that spanned the V4 region used in this study. To find overlapping OTUs between the Dorado tag OTUs recovered in this study and clones from other sites, clones that spanned the V4 region were trimmed down to cover only that region within *mothur* and were then aligned with the OTUs called from the tag data. These were then binned into OTUs at the 3% sequence dissimilarity level using *mothur*'s cluster command with the average neighbor algorithm. The percent shared OTUs was calculated by dividing the total number of shared OTUs between the Dorado tag OTUs and each site by the total number of OTUs at that respective site. For example, 196 OTUs were identified in the Santelli et al. (2008) dataset from the EPR basalts, and 115 were shared with the Dorado tag OTUs, representing 58.67% shared OTUs between Dorado and EPR—i.e., 58.67% of the OTUs recovered at EPR were also recovered at Dorado.

#### Phylogenetic tree construction

For phylogenetic tree construction, OTUs from this study, as well as the full-length sequences available from the above mentioned studies, were aligned using the *ARB-Silva* online SINA aligner (Pruesse et al., 2012) and imported into *ARB* (Ludwig et al., 2004) for identifying nearest neighbors using the SILVA Release v119 database. Where numerous OTUs from this study's tag data grouped with each other in a monophyletic clade, the most abundant OTU was selected as the representative sequence for that clade and the total number of reads for all encompassing OTUs was assigned to it. Groupings of Archaeal, Alpha-, Gamma-, and Deltaproteobacterial sequences were exported in an unaligned fasta format. GeneiousR6 (Kearse et al., 2012) was then used to align these subsets using MUSCLE (Edgar, 2004). These were exported from Geneious and, using only the near full-length reference and environmental sequences to facilitate the construction of robust reference trees, RAxML (Stamatakis, 2014) was used to generate 1000-bootstrap maximum likelihood trees under the General Time Reversible evolution model using a gamma distribution. The short tag reads from this study and short reads from the Arctic ridge study were then added to these trees using Phylogenetic Placer (pplacer) (Matsen et al., 2010). It is worth noting that because of this process, any branches solely containing these short reads from the current study or the Arctic study do not have any statistical branch support as they are simply inserted as a “best fit” within the bootstrapped maximum likelihood reference tree.

#### Accession numbers

The clone sequences recovered from this project are publicly available through NCBI′s GenBank, accession numbers KT748562–KT748628, and the raw tag data are available through NCBI′s Sequence Read Archive under project accession number SRP063681. Additionally, a fasta-formatted file containing the representative OTU sequences identified is available as a Datasheet 2 in Supplementary Material.

## Results

### Sample description

Twelve seafloor rock samples were collected from across Dorado Outcrop (Figure 1A), denoted herein as R1–R12 (R1A is a technical replicate of R1, where two different areas of the same outer rind were sampled; Table 1). The rock samples originated from two different regions of Dorado Outcrop (Figure 1A), most from areas where fluids seeping from cracks in the outcrop had elevated temperatures as compared to bottom seawater (Table 1). It should be noted the venting temperatures recorded are snapshots of venting conditions only at the time of sample collection, and returning to the same site on a different day often yielded different conditions or even no observable venting; detailed examination of these venting fluctuations was beyond the scope of this study.

Of the 12 samples recovered, one sample was a lithified carbonate (R7), and 11 of the samples (R1–R6 and R8–R12) were basalts with varying degrees of alteration ranging from highly altered, with thick (>1 cm), rough outer rinds (samples R1–R6 and R12) of a black material that resembled manganese oxides, to those with less altered, glassy, thin outer rinds (samples R8–R11). These groups will be referred to hereafter as the “more altered” (samples R1–R6 and R12) and “less altered” (samples R8–R11) sample sets. Figures 1B–D shows an example of each of these major three types (Figures S1, S3 contain images of all 12 samples). One of the rocks collected, R11, had a bright green veneer on the outside (Figure S2) that was scraped off and analyzed separately.

### DNA extraction, sequencing, and OTU clustering

The more altered basalts (*n* = 8) generally yielded more nucleic acid biomass than the less altered samples (*n* = 4), with 313 ± 190 ng DNA g^−1^ rock (mean ± 1 standard deviation) from the more altered basalts vs. 84 ± 81 in the less altered basalts (Table 2). The large standard deviations are mostly due to lone outliers in each group: R4 in the highly altered group with only 5.9 ng DNA g^−1^ rock recovered, and R10 in the less altered group with 196 ng DNA g^−1^ rock (Table 2). A Mann–Whitney (Wilcoxon rank sum) test was carried out to test the significance of this observation [due to the few samples and uneven group sizes, both of which are known to lower the power of a standard *T*-test (Zimmerman, 1987)], resulting in a *p* = 0.07.

Initial processing of the Illumina MiSeq tags resulted in 2,653,916 reads found in 72,965 OTUs (defined as 3% or less sequence dissimilarity) across the entire sample set including the two bottom water samples and the protocol blanks (*n* = 20; Table 2). The protocol blanks contained 816 OTUs, 561 of which met the criteria for removal from the sample dataset (see Section Materials and Methods). NMDS analysis of the pre-filtered dataset showed clear separation of all rock samples from the two collected bottom water samples and four extraction blanks (Figure S4), supporting our treatment of the protocol blanks' sequences. Bray–Curtis and Jaccard outputs were identical, with only minor differences in Yue–Clayton clustering (Figure S5); ultimately, Bray-Curtis dissimilarities were used in any applicable downstream analyses.

Filtering the data with a conservative 0.005% OTU abundance cutoff threshold to minimize the presence of spurious OTUs (see Section Materials and Methods) resulted in the removal of any OTU with <129 reads cumulatively (across all samples). The final data matrix used for all subsequent analyses contained 1595 OTUs comprised of 2,271,129 reads and is provided in Supplementary Materials along with a fasta-formatted file containing these representative OTU sequences (selected as the most abundant in an OTU cluster). The number of tag reads for each rock sample after processing ranged from 93,015 to 215,990 (Table 1). Despite initial DNA concentrations from the rock samples ranging across two orders of magnitude (from 4 to 600 ng DNA per gram rock), the number of OTUs per rock sample was rather consistent at 1336 ± 145 OTUs (average ± one standard deviation, *n* = 13; Table 2). The green veneer from R11 had a slightly lower OTU count of 810, whereas the bottom water samples contained <600 OTUs each.

### Basalt microbial community structure

**Figure 2:**
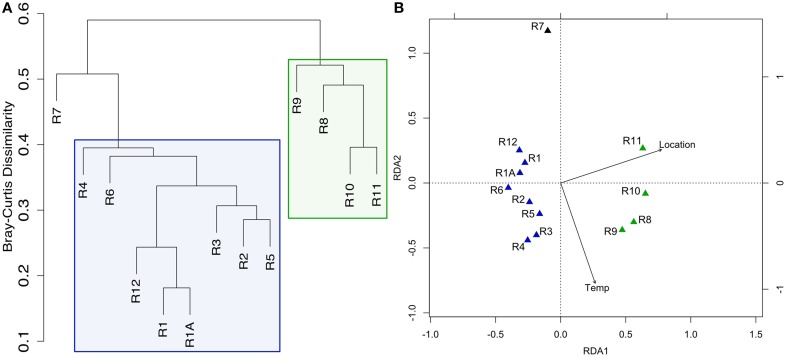
**Clustering of Dorado Outcrop sample microbial community 16S rRNA gene sequences as analyzed by (A) hierarchical clustering of community membership similarity based on the Bray-Curtis dissimilarity index and (B) redundancy analysis (RDA)**. **(A)** Microbial communities harbored by highly-altered basalt with thick Mn rinds (R1–R6, R12) group together in exclusion to less altered basalts (R8–R11), with the lithified carbonate hosting a dissimilar microbial community. **(B)** RDA showing the same clustering pattern.

Hierarchical cluster analysis and constrained ordination of the filtered basalt sequence dataset (Figures 2A,B) revealed robust groupings that statistically correlate with rock alteration characteristics and geography (although the effects of each could not be separated as alteration and geography correlate in this sample set, as discussed below). Namely, the less altered samples (R8–R11), group together to the exclusion of the more highly altered samples (R1–R6 and R12) and the lithified carbonate sample R7 (Figure 2A). As mentioned, the major dendrogram clusters and apparent levels of alteration also support a geographic relationship between the samples, as the less altered basalts were all collected from the southern end of the outcrop (Figure 1A). This end was found to be more hydrothermally active during our exploration, with more venting areas and higher temperatures detected at those areas.

Redundancy analysis supported the same sample-clustering pattern as the dendrogram (Figure 2B). The constraining variables used included location, as a binary input, for collection from the north or south end of the outcrop—a clear distinction as can be seen in Figure 1A—and the temperature of venting fluids at the time of collection (Table 1). Location was found to explain ~25% of the variance with a significant *p* = 0.002, while temperature was found to explain ~10% of the variance, however without any notable significance. An analysis of similarities test (ANOSIM, see Section Materials and Methods above) was carried out to test if the groupings by level of alteration (also being consistent with the north/south delineation) were statistically different and yielded a significant *p* = 0.001. A permutational ANOVA-test was also carried out to test the likelihood of this north/south delineation explaining the hierarchical clustering results occurring by chance; the correlation was supported (*p* = 0.0002). A second permutational ANOVA-test was run to test the ability of the observed venting temperatures at time of sample collection (Table 1) to explain the communities recovered and was also found significant, though much less so (*p* = 0.0472). However, it should be stated again that venting was observed to be variable (though consistently more active on the southern end) and these relationships should be interpreted with caution. We note that while there can be variation across the exterior of each sample (Figure S1) the grouping together of technical replicates R1 and R1A (Figures 2A,B) provides some confidence that the variation between rocks is likely stronger than the heterogeneity that may be found within one sample.

**Figure 3:**
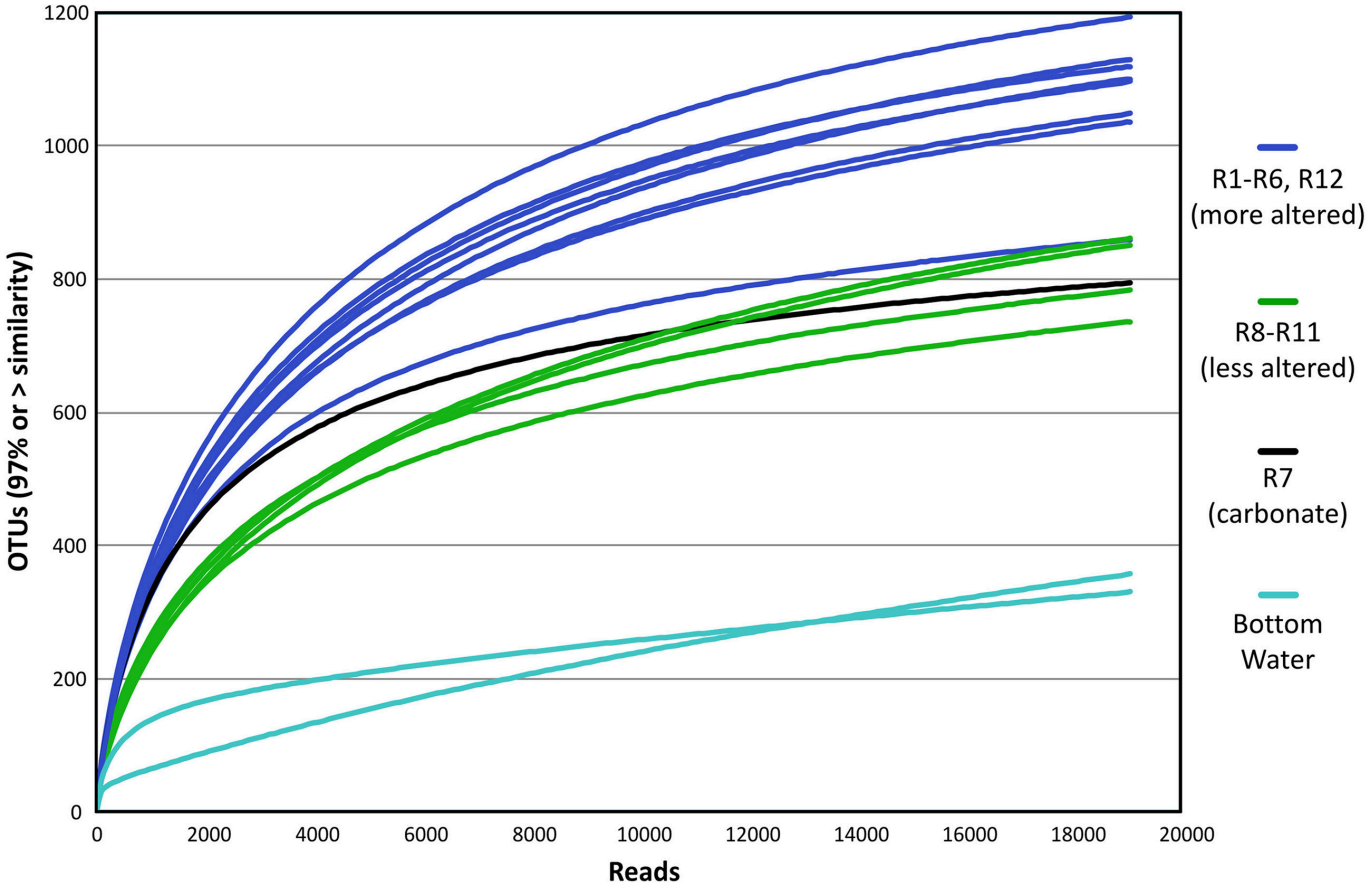
**Estimation of diversity between sample types as shown by rarefaction analysis of rock microbial community 16S rRNA gene sequence data, with each sample set subsampled to the size of the smallest dataset from bottom seawater sample**. x-Axis shows number of sequences and y-axis indicates estimated number of operational taxonomic units (OTUs) defined at the 97% or greater sequence similarity level. Blue represents the more altered basalts, green the less altered, black the lithified carbonate sample, and aqua the two bottom water samples.

To examine relative diversity between samples, rarefaction curves generated by subsampling to the depth of the sample with the least number of reads in the dataset (a bottom water sample, BW1, with 21,218 reads) show three major groupings of the samples (Figure 3). Of the observed communities retained after subsampling, the two bottom water samples were found to be much less diverse than the rock samples. And with the exception of R4, the more altered basalts (R1–R6 and R12) have distinct rarefaction curves from the less altered basalts, revealing the more highly altered basalts host more complex communities (Figure 3). A more detailed analysis of alpha diversity of the samples is not possible due to the filtering of the dataset to remove possible spurious sequences, as this eliminated any singletons and doubletons (see Section Materials and Methods).

### Major phylogenetic groups on dorado outcrop basalts

**Figure 4:**
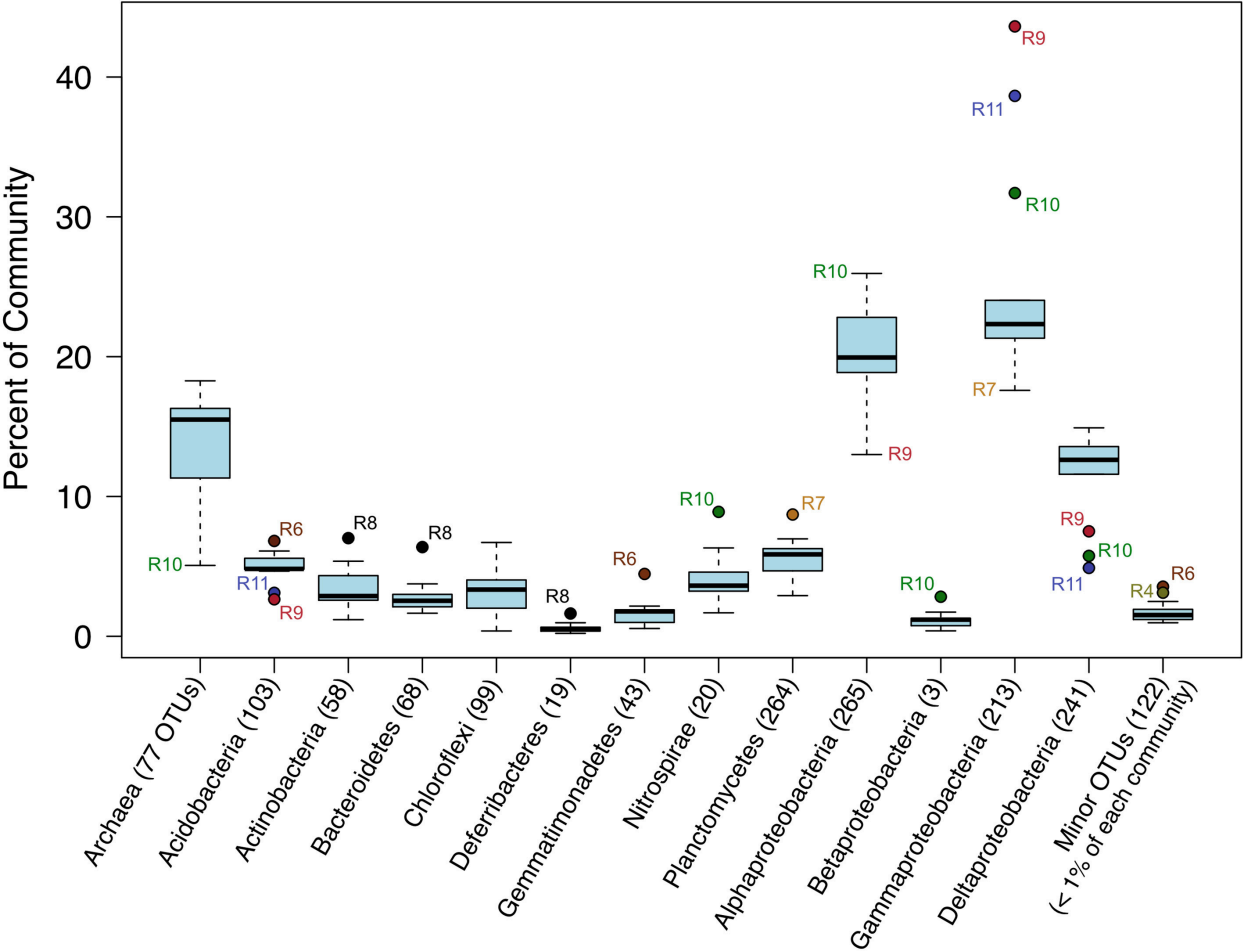
**Box-and-Whisker plot of median, upper and lower quartiles, and maximum and minimum percent abundance of 16S rRNA gene sequences from major taxa across all Dorado Outcrop basalt samples**. Outliers depicted with filled circle symbols. Number of operational taxonomic units (OTUs, defined at 97% or greater sequence similarity) in each taxonomic group indicated in parentheses.

**Figure 5:**
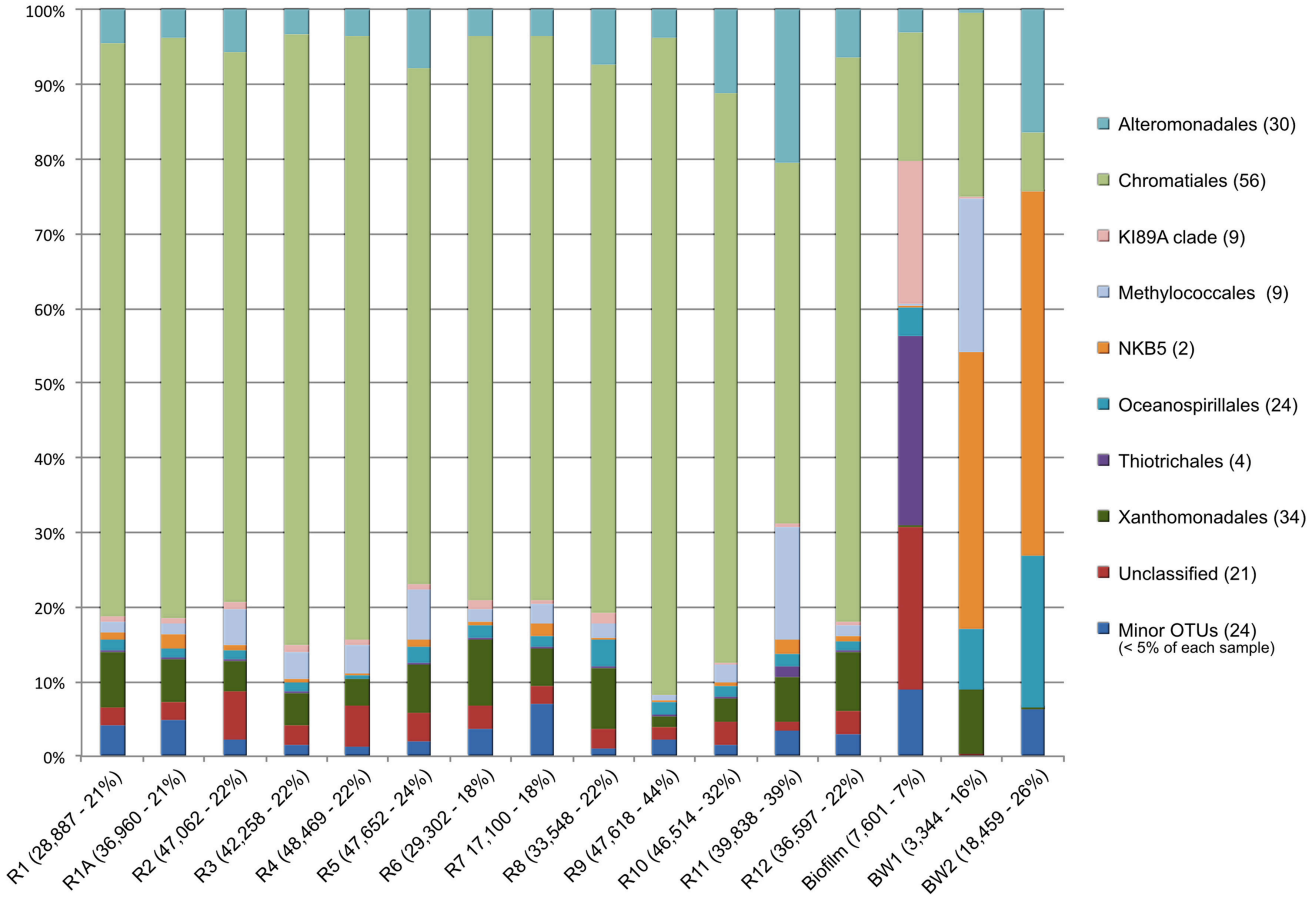
**Breakdown of Gammaproteobacteria by order**. In parentheses next to sample names on the x-axis are the total number of reads for each sample and the percentage of that sample's total they represent. In parentheses next to each taxon is the number of OTUs (97% similarity or greater) recovered in that taxon.

The relative sequence abundances of major microbial phyla were rather consistent across the basalt samples with some notable exceptions (Figure 4, Figure S6). Overall, Bacteria dominated the rock sequence libraries, making up ~86% of the observed communities. The most abundant Bacteria class on the basalts was Gammaproteobacteria, comprising an average of ~23% of sequences from each rock (Figure 4), mostly dominated by the order Chromatiales (Figure 5). The sample with the highest percentage of Gammaproteobacteria was R9 (43% of sequences), followed by R11 (38%) and R10 (31%). All three of these samples were basalts that appeared to be glassier/less altered and had thinner exterior rinds as compared to the rest of the samples collected, suggesting the less altered basalts (*n* = 4) host more Gammaproteobacteria relative to the more altered samples (*n* = 8; see Figure 1C for an image of basalt R9 and Figure S1 for all others). A Mann–Whitney U test found this difference to be significant (*p* = 0.024).

**Figure 6:**
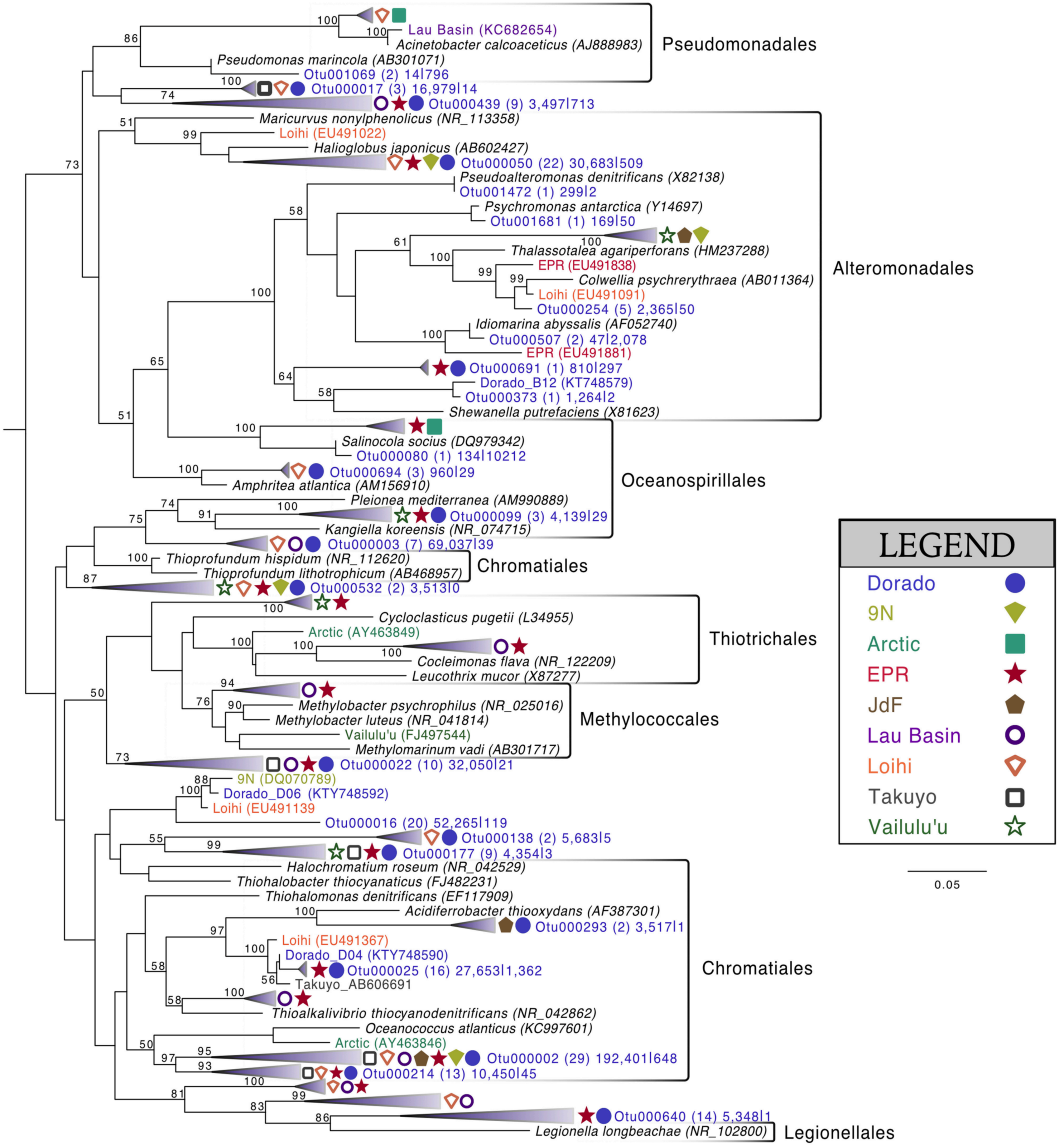
**Phylogenetic tree of seafloor basalt Gammaproteobacteria based on the hypervariable V4 region of the 16S rRNA gene**. Tree topology determined using publicly available nearly full-length sequences from environmental samples or cultivated representatives (GenBank accession number listed in parentheses), with representatives of shorter tag OTUs from this study inserted with pplacer (Matsen et al., 2010). Closely related OTUs from this study are grouped together into fans, with number of Dorado tag OTUs indicated in parentheses followed by number of sequences from all rock samples (*n* = 13) |bottom seawater samples (*n* = 2). Sequences from other seafloor basalt samples that group most closely to OTU fans indicated by symbols shown in legend. Support for branching patterns of the maximum likelihood tree from 1000 bootstraps reported at the nodes. *Mariprofundus ferrooxydans* was used as the outgroup (not shown).

Of the 213 OTUs assigned to the Gammaproteobacteria (out of 1595 total OTUs in the filtered dataset, Figure 4), 178 fall within or near the orders Alteromonadales, Chromatiales, Legionalles, Methylococcales, Oceanospiralles, Pseudomonadales, and Thiotrichales (Figure 6). The most abundant bacterial OTUs recovered (totaling ~15% of all reads collected from the rock samples) were most closely related to the obligate chemolithoautotrophic, sulfur-oxidizing *Thioprofundum* isolates within the Chromatiales as determined by BLAST sequence alignment. Representative sequences Otu000002 (192,401 sequences from rocks), Otu000003 (69,037 sequences), and Otu000016 (52,265 sequences) were 95, 96, and 93% similar to *T. lithotrophicum* and 94, 95, and 93% similar to *T. hispidum*, respectively. These species were isolated from deep-sea hydrothermal environments and are known to oxidize sulfur with oxygen and/or nitrate (Takai et al., 2009; Mori et al., 2011). These phylogenic groups are also commonly observed on basalts from other environments (Figure 6). Although the clade labeled Otu000002 in Figure 6 groups nearer to *Oceanococcus atlanticus* than to *Thioprofundum* spp., a BLAST alignment shows this OTU is only 87% similar to *Oceanococcus* as compared to 94–95% similarity to *Thioprofundum* spp. This discrepancy between BLAST alignment and tree topology is likely due to the difference between a simple pairwise alignment and using an evolutionary model as applied in tree construction (trees were built with the general time reversible model, and tests using the Jukes-Cantor model revealed the same clustering). This may also be partly due to the lower resolution resulting from the V4 region of the 16S rRNA gene; however, the near full-length EPR sequence within the compressed clade including Otu000002 was found to be 90% similar to *T. lithotrophicum*, 89% similar to *T. hispidum*, and 88% similar to *O. atlanticus*, showing this clustering is not solely an artifact of the short sequence length of the tag data (data not shown). It has been shown before that the Chromatiales are not monophyletic and splitting of this order has been supported by phylogenetic trees built with hundreds of concatenated protein sequences (Williams et al., 2010).

**Figure 7:**
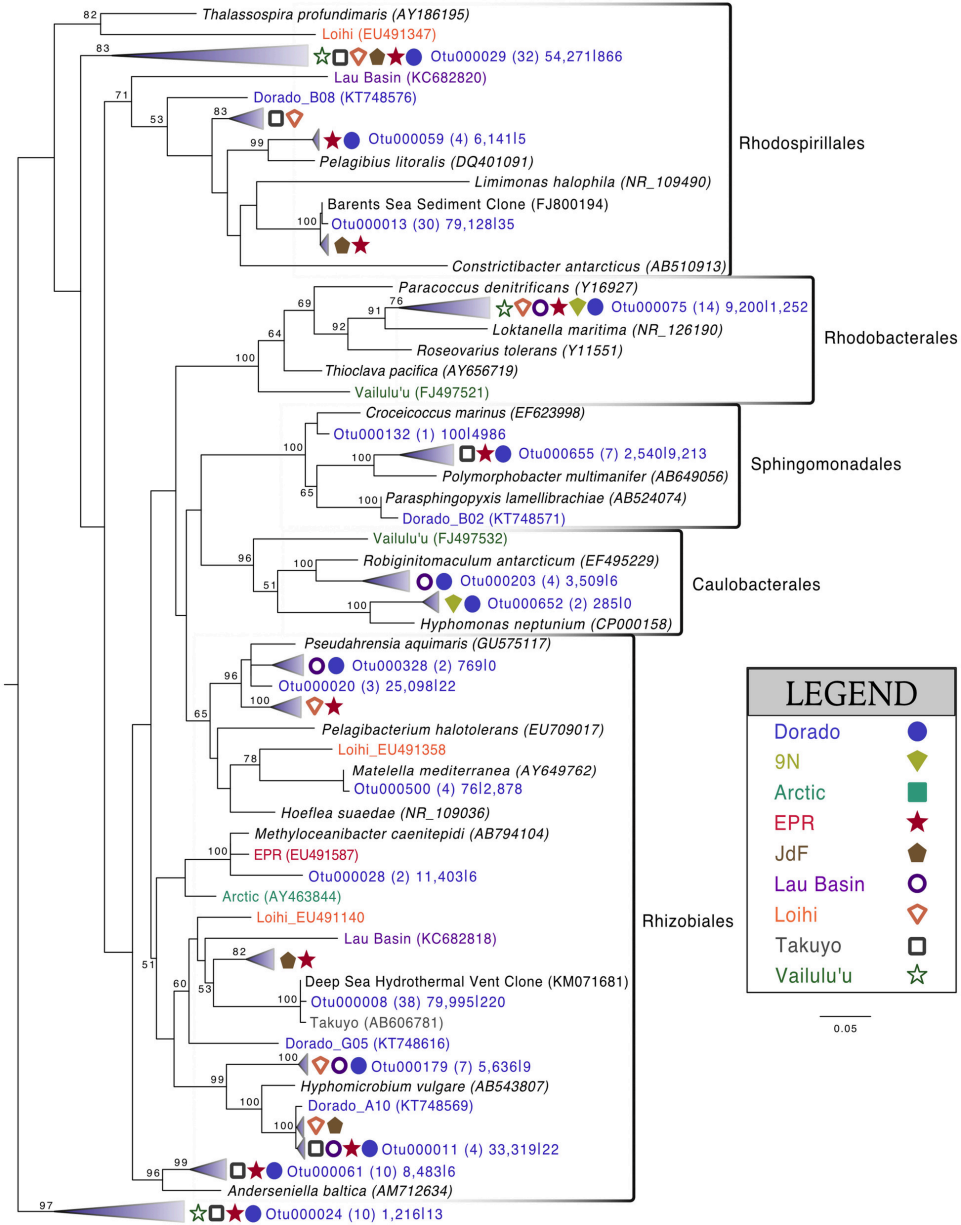
**Phylogenetic tree of seafloor basalt Alphaproteobacteria based on the hypervariable V4 region of the 16S rRNA gene, created using similar conditions as listed for Figure 6**.

Alphaproteobacteria was the next most abundant bacterial class, making up ~20% of each rock community on average, with 265 OTUs at the 3% dissimilarity level (Figure 4, Figure S6). The dominant orders identified were Rhodospirillales and Rhizobiales (Figure 7, Figure S7), with a significantly greater proportion of Rhodospirillales being found on the more altered basalts (60.5% of Alphaproteobacteria) than on the less altered (42.2%; Mann–Whitney U test, *p* = 0.006). The dominant Alphaproteobacteria groups on rock samples were also found in the water samples, though in much lower relative abundance, indicating some connectivity between these environments. The most prominent representative OTU within the Rhodospirillales, Otu000013, was found by BLAST alignment to be 99% similar to an unpublished environmental clone obtained from sediment within the Barents Sea (GenBank accession number FJ800194) and 92% similar to *Pelagibius litoralis*, an isolate from seawater off the coast of Korea (Choi et al., 2009). Otu000059 is 95% similar to the same cultured isolate. Although *P. litoralis* is a common water column bacterium, the proportion of these reads found on basalt samples as compared to the proportion recovered from the water samples grants some confidence they may be predominantly rock-hosted organisms. For example, in examining the normalized dataset, Otu000013 had over 6000 rock sample sequences and only 18 water sample sequences, and Otu000059 had 472 sequences from rock samples and only three from water samples. Otu000011 in the Rhizobiales order is 97% similar to cultured *Hyphomicrobium* spp. and is also relatively more abundant on the rocks (2500 reads or 0.12% of rock sequences) than in the water (11 reads or 0.01% of water samples).

The green veneer scraped off of sample R11 (Figure S2) was almost entirely comprised of Alphaproteobacteria (~80% of sequences, Figure S6), with the majority of sequences (~40% of total community) grouping by the SB1-18 clade (Figure S7). This Otu000024 was only distantly related (<90% similarity) to the any cultured isolates while environmental BLAST hits included 98% similarity to endosymbionts recovered from the bone-eating worm *Osedax mucofloris* collected from a whale-fall in the North Atlantic (Verna et al., 2010), and 98% similar to clones recovered from sunken wood in the Mediterranean Sea (Bessette et al., 2014). This OTU was much more rare in the rock (0.06% of sequences) and water samples (0.01%).

Deltaproteobacteria made up roughly 12% of the rock communities on average, with 241 OTUs being called at the 3% dissimilarity level (Figure 4), with sequences grouping within the Myxococcales, Desulfobacterales, Syntrophorhabdaceae, Desulfarculales, and Desulfuromonadales (Figures S8, S9). The same group of less altered basalts that had a significantly higher proportion of Gammaproteobacteria as compared to the more altered samples also had a lower proportion of Deltaproteobacteria (Figure 4), though not found to be significantly different when including R8 in the test (Mann–Whitney U test, *p* = 0.11).

Major bacterial groups recovered outside of the Proteobacteria phylum included, by order of relative abundance (Figure 4), Planctomycetes, Acidobacteria, Nitrospirae, Actinobacteria, Gemmatimonadetes, and Chloroflexi, all of which have been identified in basalt samples from previous studies (Lysnes et al., 2004; Mason et al., 2009; Nitahara et al., 2011; Sylvan et al., 2013). All together these make up <20% of each community of the rocks sampled.

**Figure 8:**
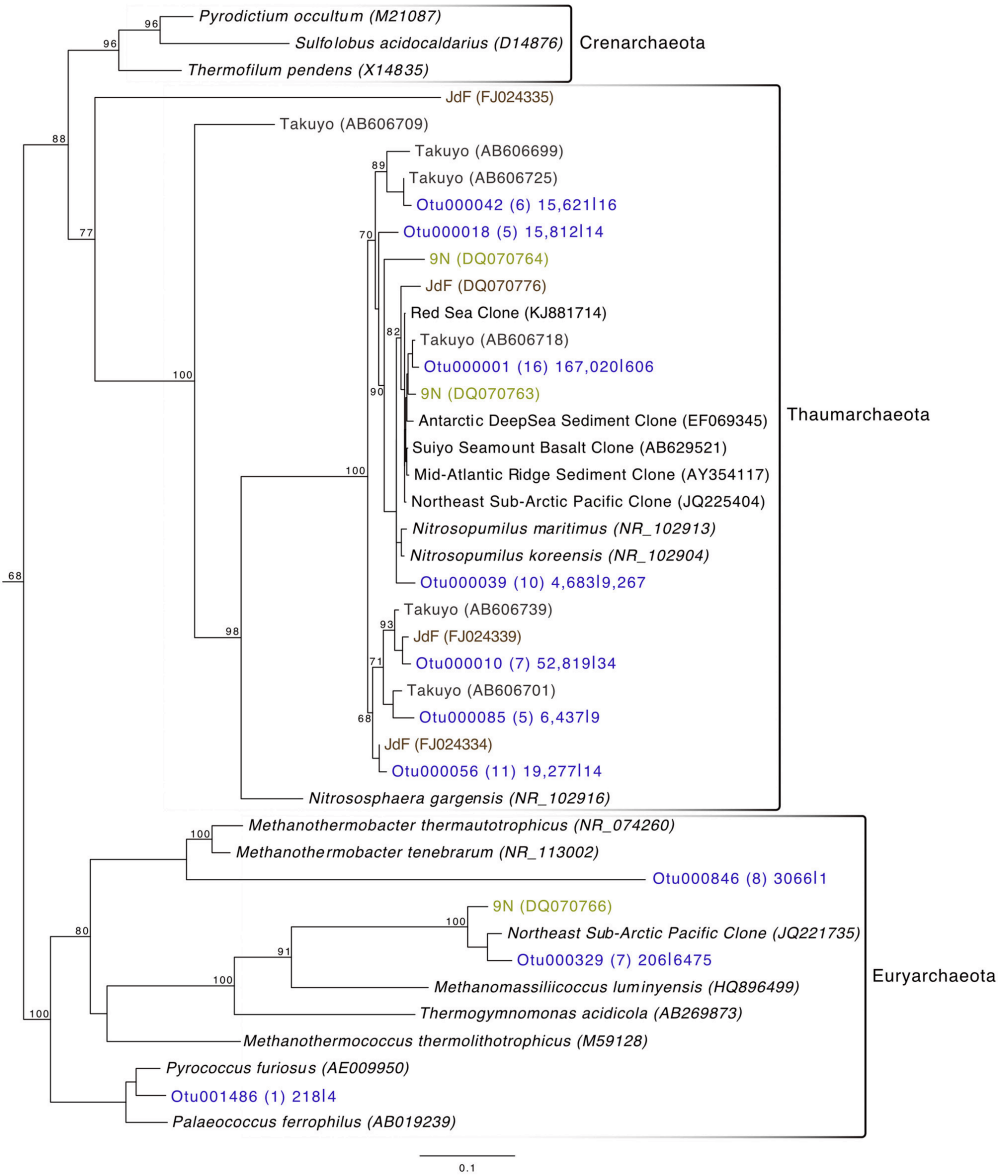
**Phylogenetic tree of seafloor basalt Archaea based on the hypervariable V4 region of the 16S rRNA gene, created using similar conditions as listed for Figure 6**.

Archaeal reads averaged about 15% of total communities on the basalts collected, with significantly fewer being found on the less altered samples (an average of 8.5%) than on the more altered basalts (15.7%; Mann–Whitney U test, *p* = 0.02). Though Bacteria overall are more dominant than Archaea, the most abundant OTU in the entire dataset was archaeal. Otu000001 (167,020 sequences from rocks) groups near the cultured Marine Group I Thaumarchaeota *Nitrosopumilus maritimus* and *Nitrosopumilus koreensis* (Figure 8), with BLAST alignments of 97% similarity to both. Members of this genus are globally-distributed, known chemolithoautotrophic ammonia-oxidizers suspected to play an integral role in carbon and nitrogen cycling (Walker et al., 2010). While the genus *Nitrosopumilus* is ubiquitous in deep marine waters, some of the OTUs appear to have a preference for basalts as opposed to water. For example, Otu000001 represents 8.1% of the total reads recovered from the rock communities, and only 0.7% of the water communities, whereas, for example, Otu000039 that groups closer to *Nitrosopumilus* spp. represents 0.2% of the rock communities and 10% of the water samples.

### Similarity of dorado outcrop basalt communities to other basalt samples

Comparison of the Dorado Outcrop tags and clone library sequences with other previously published basalt sample clone datasets revealed several monophyletic clades of basalt-derived sequences. For example, within the Gammaproteobacteria, the collapsed clade labeled as Dorado tag Otu000002 (Figure 6) contains clones from six out of the eight other sites included in the analysis (the only exceptions being the young Vailulu'u seamount and the Arctic Ridge, which has a clone very near). This was the most abundant bacterial OTU recovered in this study, totaling over 10% of all reads collected, and is only 95% similar to the cultured obligate chemolithoautotrophic, sulfur-oxidizing *Thioprofundum* spp. As another example, within Rhodospirillales of the Alphaproteobacteria, Otu000029 contains sequences from Takuyo, Loihi, JdF, and EPR, with a Vailulu'u clone branching nearby (Figure 7). This representative Dorado tag OTU, Otu000029, contained about 2.5% of all sequences recovered from the 12 rock samples and has only 93% sequence similarity to *Limimonas halophile*, a chemoheterotroph isolated from a hypersaline lake (Amoozegar et al., 2013). Together with closely related Otu000013, these two OTUs make up over 6.5% of total sequences recovered in this study, and they have near neighbors that were recovered from five out of the eight other sites examined, suggesting a global distribution of this clade. Another Alphaproteobacteria clade containing Otu000075 within the Rhodobacterales also has clones from Vailulu'u, Loihi, Lau Basin, EPR, and 9N. These are most closely related to the cultured representative *Loktanella maritima* (96% similarity), which was recently isolated from shallow sediments in the Sea of Japan (Tanaka et al., 2014). Within the Rhizobiales order there are numerous clades branching out around the cultured *Hyphomicrobium vulgare*, containing sequences recovered from every site except the very young Vailulu'u and 9N samples (being from recent eruptions about 10 and 20 years ago, respectively). Within the Deltaproteobacteria, a large monophyletic clade is found between the orders Desulfarculales and Desulfuramonadales that contains basalt sequences from many sites (Figure S8) with no closely related cultured representatives (<86% similarity to *Geobacter* spp.). Similarly, another basalt clade found within the Deltaproteobacteria is represented by Otu000057 and has near neighbors from Loihi, EPR, Takuyo, and the Atlantic Arctic Ridge. Additionally, a large clade of Thaumarchaeota comprised of basalt and sediment environmental sequences was observed grouping near *Nitrosopumilus* spp. (Figure 8).

**Figure 9:**
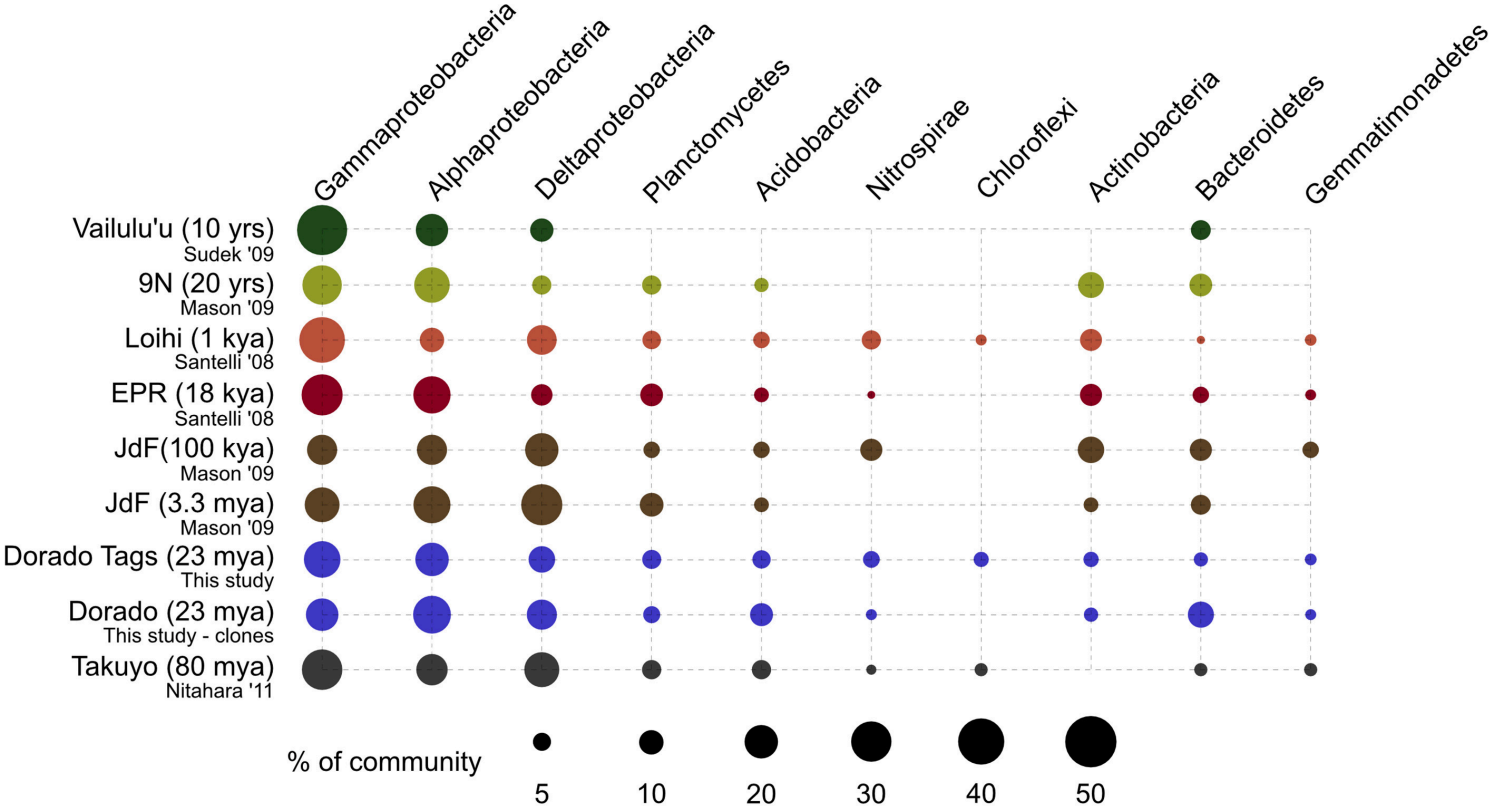
**Comparison of major bacterial taxa abundances between Dorado Outcrop rocks and other seafloor basalt sample sets**. Proportions of 16S rRNA gene sequences in each taxonomic group indicated by size of circles, as shown in legend. Dorado Outcrop dataset from tag sequencing of V4 hypervariable region, while other sample sets are clone library data of nearly full-length 16S rRNA gene sequences. Ages of samples sets are listed in parentheses. From top to bottom: *n* = 142, 35, 470, 352, 25, 32, 2,068,892, 67, 86.

**Figure 10:**
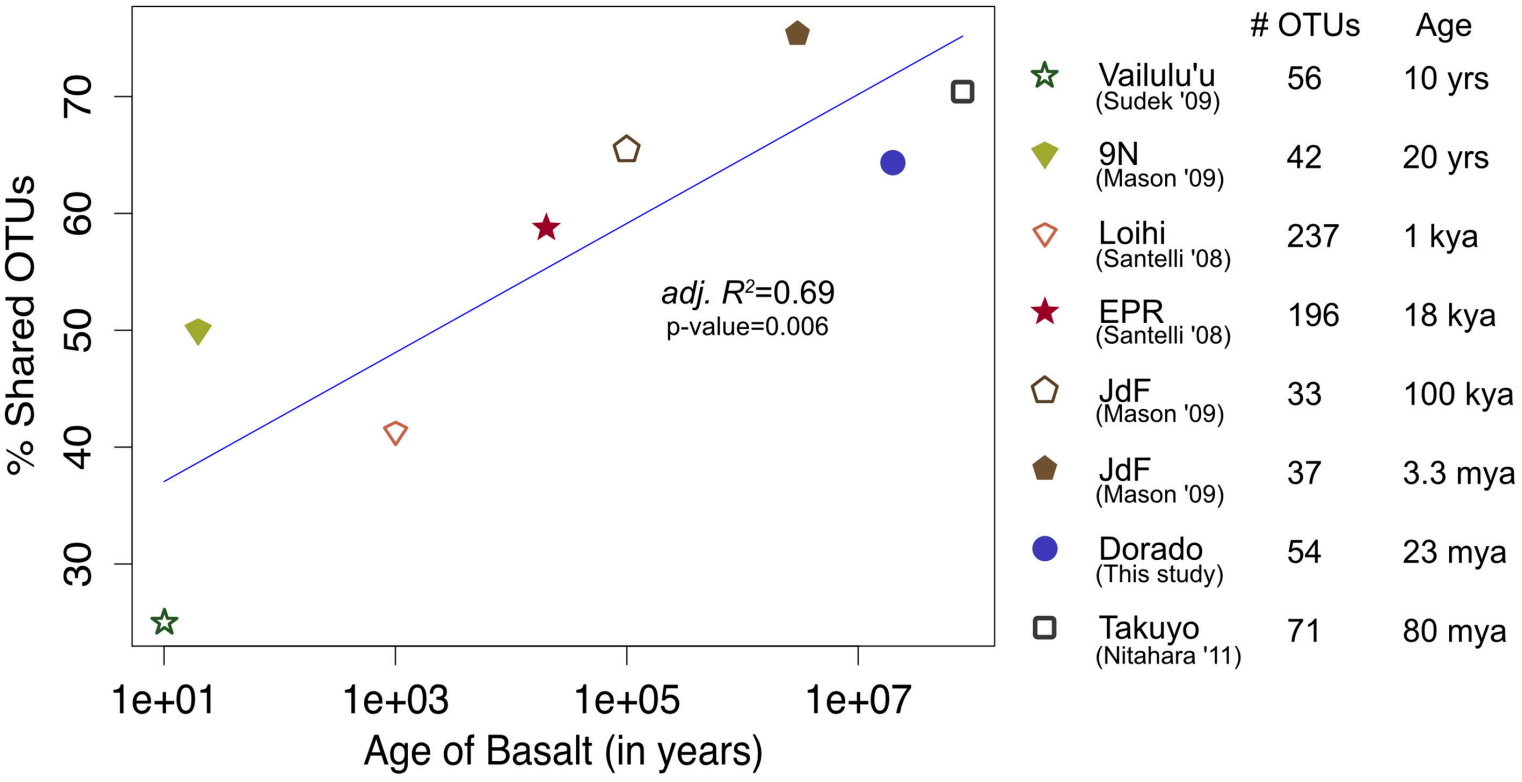
**Bacterial community overlap between Dorado Outcrop 16S rRNA gene V4 hypervariable region sequence OTUs from this study in comparison to clone library sequences from other seafloor basalt samples trimmed to the same region, as based on percent of shared operational taxonomic units (OTUs) defined at the 97% or greater sequence similarity level, and plotted in relation to site age**. From top to bottom in the legend, number of samples available from each study that contributed to the clone libraries: 1, 1, 4, 6, 1, 1, 1, 1.

At a broader taxonomic level, all basalt samples examined in this study, as well as previous studies, are dominated by Gamma-, Alpha-, and Delta-proteobacteria (Figure 9). In alignment with results of this study, wherein the less altered basalts were found to have a greater proportion of Gammaproteobacteria and lower proportion of Deltaproteobacteria than in the more altered samples (Figure 4), the proportions of Gammaproteobacteria appear to decrease while Deltaproteobacteria increase as age increases in basalts (Figure 9). Additionally, Bacteroidetes have been recovered from every site, as were Planctomycetes and Acidobacteria (with the exception of the very newly erupted Vailulu'u sample), whereas other bacterial phyla do not have consistent trends. However, sequence library size may play a factor in this interpretation, with sample sets ranging from only 32 clones up to 470. The use of different DNA extraction methods, primer sets, and sequencing technologies may also skew these interpretations. As one measure of this, the single clone library constructed from Dorado sample R5 was compared to the Dorado tag sequence library. Only 61% of OTUs identified in the clone library (33 out of 54 total clone OTUs) were shared with OTUs from the tag library, even though these were from the same site. It should be noted this may also be due to the tag dataset having already been clustered into OTUs at the 3% dissimilarity level. While these differences prohibit any quantitative analyses between sites, it still allows a conservative window into the presence or absence of specific OTUs globally. Following these caveats, an analysis of the Dorado Outcrop basalt tag OTU library with clone libraries from other geographically-disparate studies reveals a significant trend of an increasing proportion of shared OTUs with sample set age (*p* = 0.006; Figure 10).

## Discussion

As a low-temperature (5–15°C) venting site likely representative of millions of similar features on the seafloor (Etnoyer et al., 2010; Wessel et al., 2010), Dorado Outcrop represents a new end-member in the global survey of hydrothermal systems and basalt biomes that has heretofore not been examined. Our analysis of 16S rRNA genes from Dorado Outcrop basalts confirms the pattern of overall basalt microbial community dominance by Gamma-, Alpha-, and Deltaproteobacteria (Figure 4), consistent with previous surveys of much younger and older basalts from geographically distant locations (Figure 9). Furthermore, this new sequence study provides further support for the idea put forward previously (Mason et al., 2009) that there is a clade of basalt-hosted Thaumarchaeota very closely related to the genus *Nitrosopumilus* (Figure 8). There also appears to be an age correlation in the overlap of microbial community membership, with increasing similarity of communities with age (Figure 10), suggesting that species endemism on young basalts possibly diminishes with aging, yielding to a shifting community dynamic that may eventually stabilize to reflect a more global seafloor basalt biome.

### Diversity and potential function of microbial communities on dorado outcrop basalts

The majority of sequences recovered were bacterial, dominating samples about 85–15% over Archaea (Figure 4). Previous studies have ranged from finding <0.02% Archaea (*n* = 7) through qPCR on samples dredged up from the Knipovich Ridge up in the Atlantic–Arctic Ocean (Einen et al., 2008), to 4–12% (*n* = 5) on samples collected directly from the seafloor at the EPR (Santelli et al., 2008), to 65% (*n* = 1) on a sample collected from the seafloor at Takuyo Seamount near Japan, though clone libraries from the same study yielded 17% Archaea with the discrepancy suspected by the authors to be due to PCR bias (Nitahara et al., 2011). The Arctic Ridge samples varied in age from <30 years to 100 kya, the EPR samples were estimated at <18 kya, and Takuyo at ~80 mya. Due to the overlap in age between the Arctic and EPR samples, a simple correlation with age to explain the drastic shift in proportion of Archaea recovered does not suffice. However, data from this study suggest that more altered basalts host a higher proportion of Archaea than those that are less altered (16.7% as compared to 8.5%, Mann–Whitney U, *p* = 0.02).

The most abundant individual OTU recovered across all rock samples in the current study (making up just over 5% of all reads recovered) was comprised of sequences representative of Thaumarchaeota most closely related to two isolates of the *Nitrosopumilus* genus. Though *N. maritimus* is commonly found in the water column (Karner et al., 2001), *N. koreensis* was isolated through enrichment cultures of Arctic sediment (Park et al., 2010). And while 16S rRNA gene similarities (99%) group these species very near each other (Figure 8), genomic analysis has identified that outside of core genes involved in ammonia oxidation and lithotrophy, 30% of *N. koreensis's* genes are unique with respect to *N. maritimus*, demonstrating these species may possess more differences than their ribosomal phylogeny would suggest alone (Park et al., 2012). This gives confidence to the idea of a crust-specific clade of *Nitrosopumilus*, just as *N. koreensis* appears to be a sediment-specific clade. We suggest that Otu000001 (Figure 8) represents a rock-associated *Nitrosopumilus* as it comprises 8.1% of the rock communities recovered, and only 0.7% of the water communities, whereas, in contrast, Otu000039 makes up only 0.2% of the rock communities, and 10% of the water communities. Given their abundance and global distribution as chemolithoautotrophic ammonia-oxidizers, it is likely these organisms play a key role in C and N cycling in the global basalt biome in addition to the sediment and water column.

It is likely that S and C cycling, and possibly N cycling, are partially mediated by the highly abundant OTUs that are most closely related to *Thioprofundum lithotrophicum* and *Thioprofundum hispidum* (95–96% similar) within the Gammaproteobacteria. These are known chemolithoautotrophic sulfur-oxidizers that utilize nitrate and oxygen as electron acceptors, and they are commonly observed on globally distributed basalts (Figure 6). *Thioprofundum* group within the Chromatiales order, which has previously been indicated as a group involved in carbon fixation on seafloor basalts via the Calvin cycle using RuBisCO form II (Orcutt et al., 2015). Microarray analysis of a seafloor basalt sample from JdF revealed RuBisCO genes (Mason et al., 2009), and quantitative PCR of globally distributed basalts revealed a higher abundance of Calvin cycle genes compared to other carbon cycling pathways (Orcutt et al., 2015). Thus, we propose that the *Thioprofundum*-related OTUs observed in this study, and commonly observed on basalts, are likely major contributors to carbon cycling on basalts, gaining energy from the oxidation of reduced sulfur with either oxygen or nitrate. Although Deltaproteobacteria are commonly known to be involved in sulfur cycling in the marine environment, sequences recovered from this study were only 86% similar to cultivated species, making any assignment of function tenuous. Relative abundance of the Deltaproteobacteria indicates that they may be restricted to anaerobic microniches within the basalt rind that builds up over time, since their sequences are more abundant in altered basalts (~13% of sequences) than on less altered basalts (~8%), and since sequence abundance seems to be lowest on the youngest basaltic substrates (Figure 9).

Many of the basalts recovered in this study had thick manganese oxide rinds with visible iron oxide staining, indicating that manganese and/or iron cycling may support chemolithotrophy in these samples. Within the Alphaproteobacteria, the relatively abundant Otu000011 (with 1.5% of total sequences from rocks) is 95% similar to *Hyphomicrobium vulgare*, a known manganese oxidizer. Within the Gammaproteobacteria, Otu001472, though low in abundance, is 100% similar to the known manganese oxidizer *Pseudoalteromonas denitrificans* (Figure 6). Observation of these groups indicates the possibility of Mn oxidation occurring on the basalts. Otu000652, within the Alphaproteobacteria, groups near the known iron-oxidizer *Hyphomonas neptunium* at 96% sequence similarity (BLAST alignment; Figure 7). Possible iron-reducers can be found within the Alteromonadales clade, with Otu000373 closely related to *Shewanella putrefaciens* (97% similarity according to BLAST alignment; Figure 6). Notably, no sequences from the known marine neutrophilic iron oxidizing Zetaproteobacteria, commonly found in areas with relatively high concentrations of dissolved reduced iron (Emerson et al., 2007), were detected in this study. Thus, the identities of potential iron oxidizing bacteria on the Dorado Outcrop basalts are unclear, as it often the case in other studies of basalt microbial communities (Mason et al., 2007, 2009; Santelli et al., 2009).

### Biogeography of clades enriched on basalt

As has been investigated previously (Mason et al., 2007, 2009), phylogenetic trees constructed with 16S rRNA gene sequences recovered from multiple studies of geographically distinct seafloor basalts, nearest cultured and environmental samples, and this study elucidate many lithic clades that appear ubiquitous in their distribution. One example includes the highly abundant Gammaproteobacteria most closely related to members of the genus *Thioprofundum* represented by Otu000002 (Figure 6). The abundance of these sequences recovered from Dorado Outcrop and its presence in almost every other dataset support that this is likely a globally-significant basalt ecotype that may play a role in C, S, and potentially N cycling at the seafloor. Similarly, several monophyletic clades consisting of basalt sequences from all sites examined can be found within the Deltaproteobacteria, e.g., those surrounding Otu000027, Otu000037, Otu000057, and Otu000074 (Figure S8). Furthermore, all basalt studies that have recovered Archaeal sequences (Thorseth et al., 2001; Lysnes et al., 2004; Mason et al., 2007, 2009; Nitahara et al., 2011) have observed the Thaumarchaeota clade that has been suggested to be ocean-crust specific (Mason et al., 2009), and that is the single most abundant OTU in the Dorado Outcrop dataset (Figure 8). Considering the close sequence similarity of this clade to *N. maritimus* and *N. koreensis*, it is likely these organisms play an important role in ammonia oxidation and C cycling at the seafloor. While this clade is also found in the water column, the much higher relative abundance of this group in the basalt samples suggested that it has a lithic niche. Supporting the idea of lithic niches for *Nitrosopumilus*-related groups, subaerial ferromanganese deposits in terrestrial caves have been found to host abundant Thaumarchaeota (Northup et al., 2003) with 86–90% sequence similarity to *Nitrosopumilus* and Otu000001 and Otu000010 from this study.

The common appearance of such abundant clades on globally distributed seafloor basalts begs the question of what drives these similarities. The relationship of increasing diversity correlating with increasing levels of alteration has been put forward before (Lysnes et al., 2004; Mason et al., 2009; Santelli et al., 2009), and this premise is supported by rarefaction analysis of the Dorado Outcrop basalts (Figure 3). In many cases, such as in the present study, detailed chemical composition data of the basalts is not available, preventing an analysis of how much rock composition drives these similarities. As a proxy for rock composition and alteration state, we examined the similarity of basalt microbial communities based on the approximate age of the basalts. By calculating the percent of shared OTUs between this study's tag data and other sites' clones to further investigate any presence/absence relationships of specific OTUs, a strong positive correlation between percent OTUs shared and increasing age of basalt emerged (adj. *R*^2^ = 0.69, *p* = 0.006, Figure 10). However, the largest difference, and much of the support of the regression, seems to be due to the three youngest data points (from Vailulu, 9N, and Loihi); when these sites are removed from the analysis, the correlation is no longer significant (adj. *R*^2^ = 0.10, *p* = 0.31, data not shown). Also, although the regression based on age is strong (*R*^2^ = 0.69) with a significant relationship (*p* = 0.006), the low number of available sequences from some samples/sites and the methodology of comparing tag sequence data to clones further convolutes this apparent relationship to an unknown degree. For example, when performing the same analysis with only the clone data from this study (*n* = 67 sequences) compared to the clones from other sites, the correlation disappears (data not shown). This is likely due to insufficient data as a result of the relatively shallow depth offered by clone libraries. Nevertheless, while the correlation of shared communities with regard to age requires deeper examination, the percentage of OTUs from other sites that were also observed at Dorado Outcrop ranges from 25 to 75%, with more overlap being found at older sites.

Due to Dorado being 23 million years old and having the most overlap with “older” sites (>1000 years), this suggests there may be a broader relationship underlying the available data wherein very young, glassier basalts (<1000 years old in this case) host unique communities as compared with those >1000 years old. Pitting of fresh, glassy basalt surfaces as a result of biological activity has been observed in the lab to occur within weeks, showing fresh basalts can be, and likely are, immediately colonized after formation at the seafloor (Staudigel et al., 1995, 1998; Thorseth et al., 1995). This initial environment, composed solely of newly solidified basalt conditions, may provide only a relatively narrow range of distinct habitats for microorganisms. Aged basalts, in contrast, accrete organics and allochthonous, biologically-significant metals during the course of abiotic/biotic alterations, and develop stratified outer rinds with cracks and fissures providing aerobic/anaerobic microniches with a greater availability of energy sources (Mero, 1962; Zinger et al., 2012). Thus, they may come to host extremely diverse, though globally ubiquitous, communities.

## Conclusions

This work provides the first community assessment of a new basalt biome—with regard to age and hydrothermal properties—that is likely representative of millions of outcrops globally. In alignment with previous studies, Gamma-, Alpha-, and Deltaproteobacteria seem to dominate the lithic communities, and greater biodiversity was found on basalts with more highly altered outer rinds. We identified several shared OTUs between global seafloor basalt studies that appear to form monophyletic clades to the exclusion of other environments. This conservation of certain taxa colonizing seafloor basalts is likely due to the selective geochemical environment and to there being virtually no dispersal limitation for microbes throughout the oceans. Of these globally ubiquitous clades, particularly of note are abundantly recovered OTUs from this study that are closely related to sulfur-oxidizing, chemolithoautotrophic Gammaproteobacteria, as well as what appear to be crust-specific ammonia-oxidizing archaea. The cosmopolitan distributions, high proportions, and lineages of these organisms suggest they may play substantial roles in C, N, and S cycling globally, and identifies them as clear subjects for future, targeted metagenomic and metatranscriptomic efforts. Our analysis also reveals a significant correlation between basalt age and microbial community membership. From the limited available data, it appears that upon initial formation basalts host distinct communities as compared to those greater than a few thousand years old. Moreover, it appears the communities may stabilize somewhat over time as the proportion of shared OTUs between Dorado (23 mya) did not greatly fluctuate between those that are ~18 kya to a site that is ~80 mya. It seems as though the initial environment, driven primarily by only those substrates sourced from the basalt itself, selects for a founding population that over time begins to shift, giving way to a much more diverse lithic community. Mechanisms that may be involved with driving this shift include the persistent accretion of metals and organics through fluid-deposition, the accumulation of breakdown products, and the spawning of various microniches coincident with ongoing biotic/abiotic weathering and development of an outer alteration rind that is ultimately likely to be more indicative of the global seafloor basalt biome.

## Author contributions

BO, KE, and ML conceived the study; BO and ML collected the samples; and ML carried out all laboratory analyses. ML performed all data analysis with support from NW and JS. ML wrote the manuscript with input from all authors.

### Conflict of interest statement

The authors declare that the research was conducted in the absence of any commercial or financial relationships that could be construed as a potential conflict of interest.
