# Supplementary material for: Microbial Communities on Seafloor Basalts at Dorado Outcrop Reflect Level of Alteration and Highlight Global Lithic Clades
Source: Front Microbiol. 2015 Dec 23;6:1470. doi: 10.3389/fmicb.2015.01470 (PMC4688349; doi:10.3389/fmicb.2015.01470)
Supplement: Supplementary file 3 [file Image1.PDF]

Supplementary Material

Microbial communities of seafloor basalts at Dorado Outcrop reflect level of alteration and highlight global lithic clades

Michael D. Lee, Nathan G. Walworth, Jason B. Sylvan, Katrina J. Edwards, Beth N. Orcutt*

*** Correspondence:** Beth N. Orcutt: borcutt@bigelow.org

1 Supplementary Figures

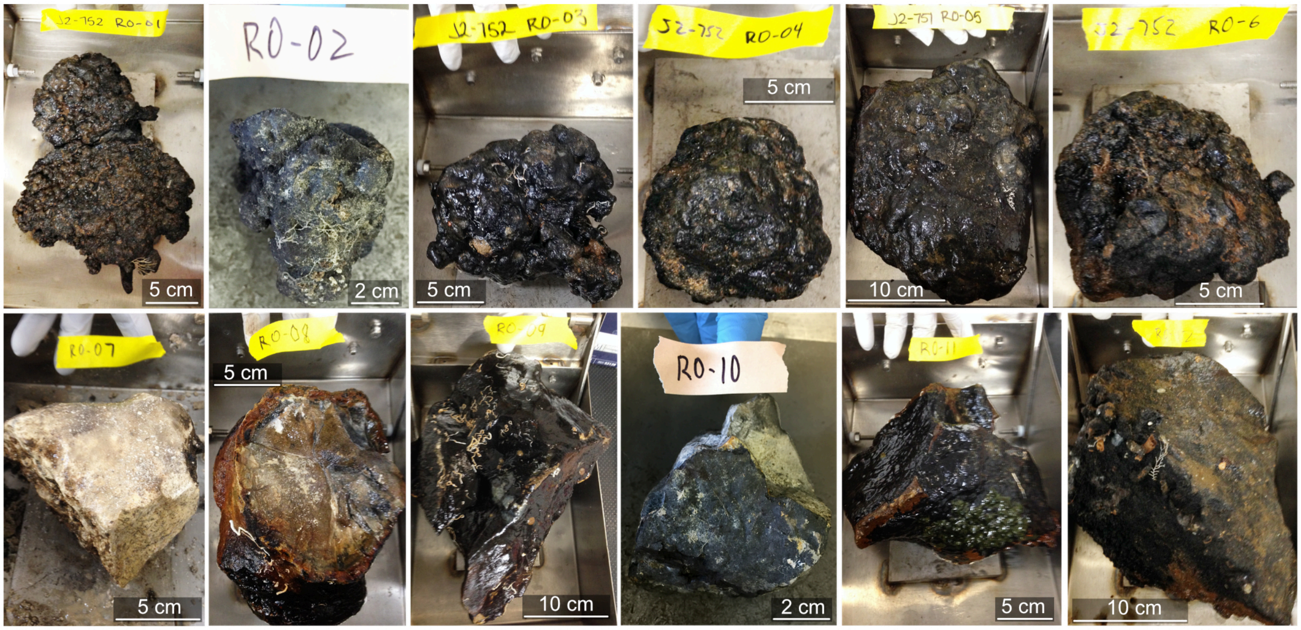

Figure S1. Photographs of all 12 rock samples analyzed in this study. Photographs by Michael Lee.

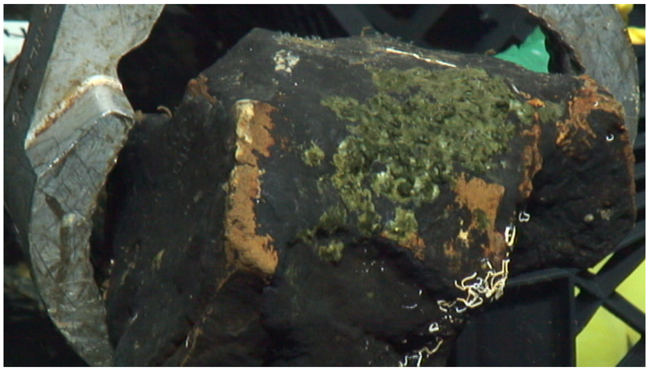

Figure S2. Seafloor photograph of the green veneer on the surface of rock sample R11 taken with ROV *Jason-II* (courtesy of the Woods Hole Oceanographic Institution, cruise AT26-09, chief scientist Geoff Wheat).

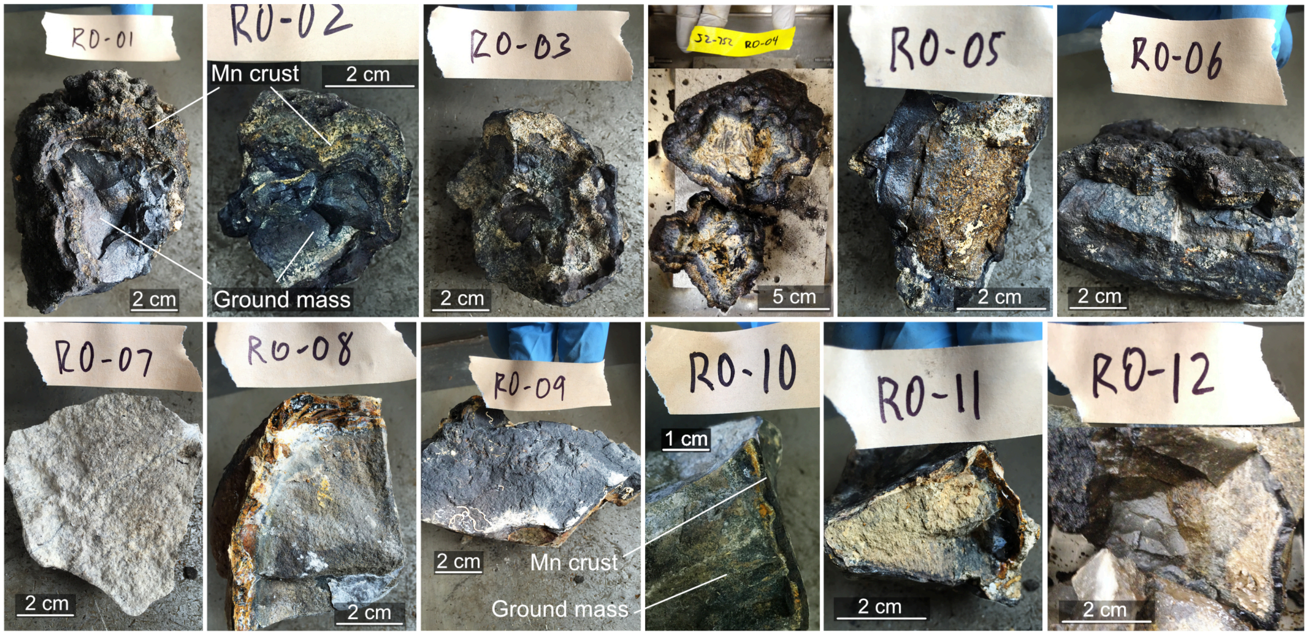

Figure S3. Photographs of all 12 rock samples analyzed in this study with the outer rind and ground mass easily discernable in some. Photographs by Michael Lee.

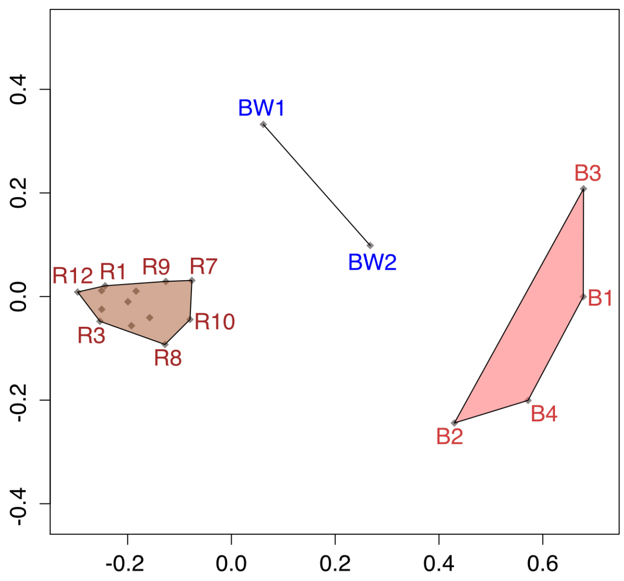

FIGURE S4: Non-metric multidimensional scaling ordination showing microbial communities from rock samples (labeled with R#) clearly separate from bottom seawater microbial communities (labeled BW#) and from contaminant sequences in DNA extraction blanks (labeled B#).

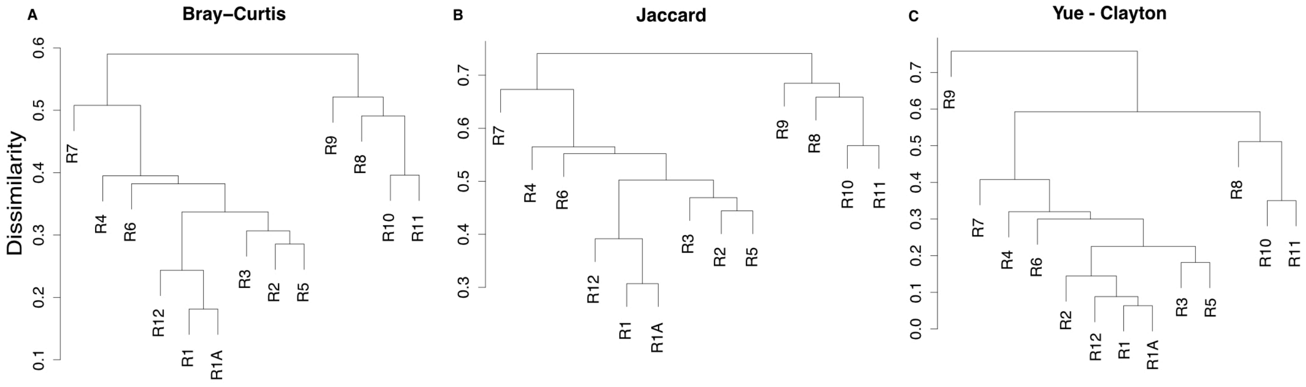

Figure S5. Hierarchical clustering of all rock samples with Bray-Curtis, Jaccard, and Yue-Clayton dissimilarity indices.

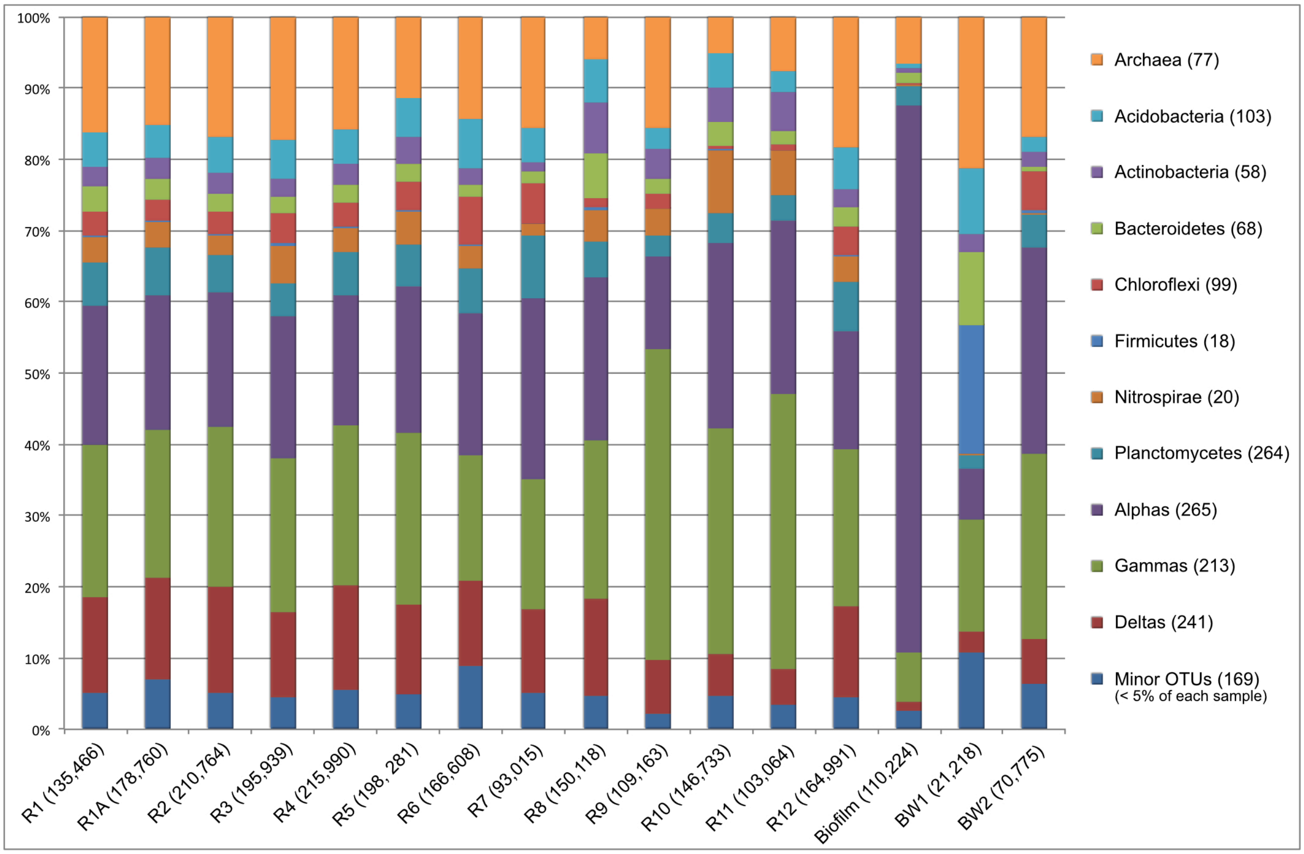

Figure S6: Breakdown of major taxa. In parentheses next to sample names on the x-axis are the total number of reads for each sample. In parentheses next to each taxon is the number of OTUs (97% similarity or greater) recovered in that taxon.

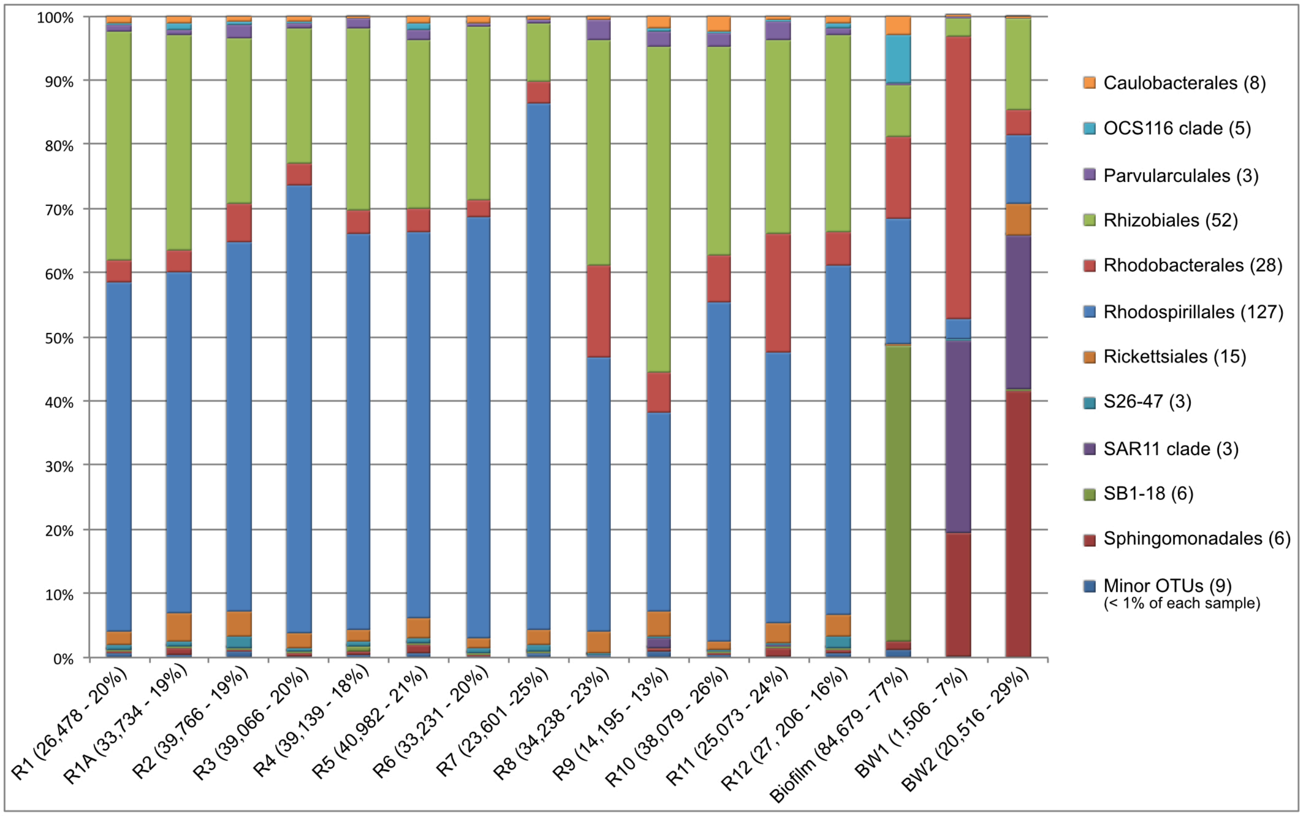

Figure S7: Breakdown of Alphaproteobacteria by order. In parentheses next to sample names on the x-axis are the total number of reads for each sample. In parentheses next to each taxon is the number of OTUs (97% similarity or greater) recovered in that taxon.

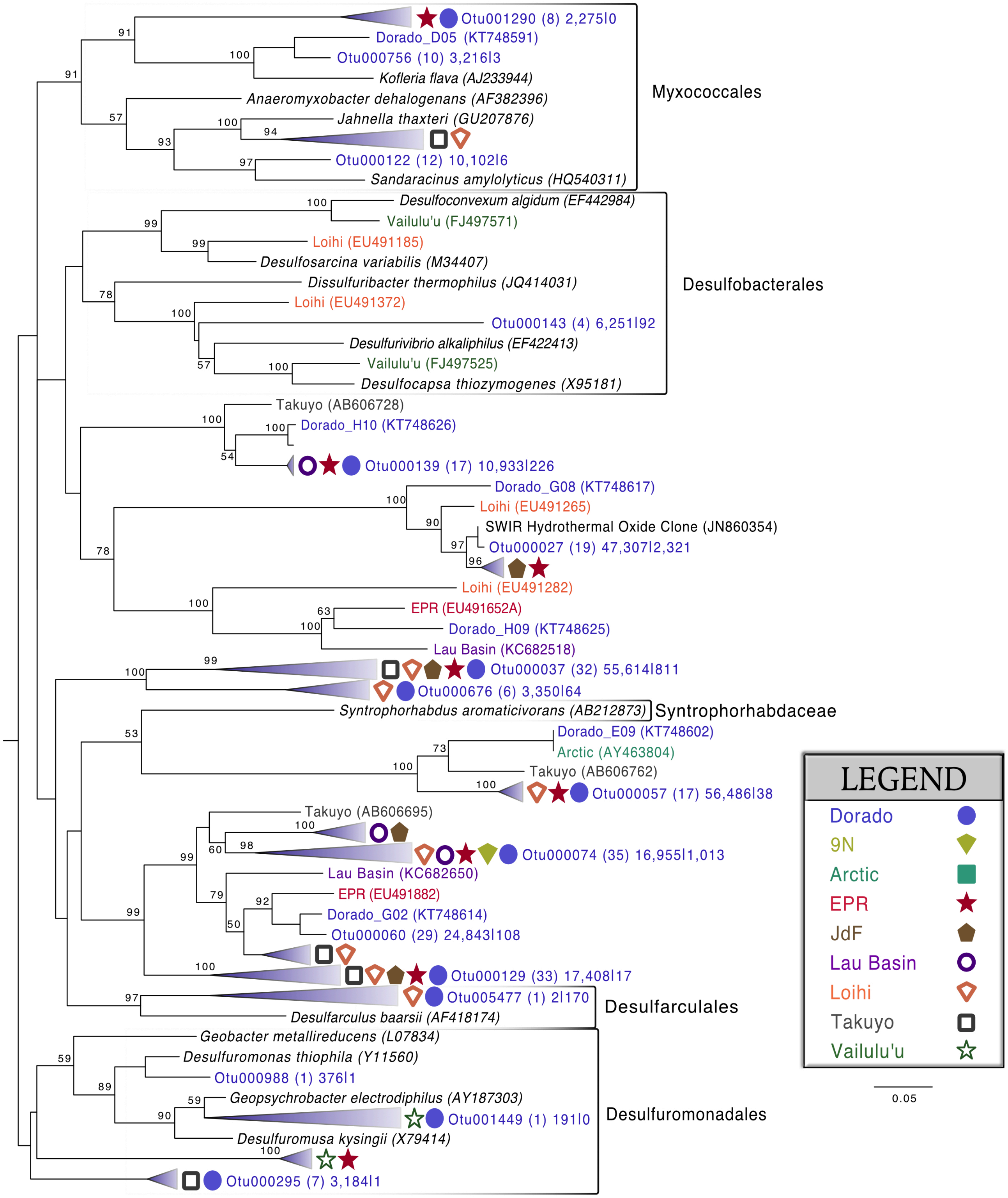

Figure S8. Phylogenetic tree of seafloor basalt Deltaproteobacteria based on the hypervariable V4 region of the 16S rRNA gene, created using similar conditions as listed for Figure 6.

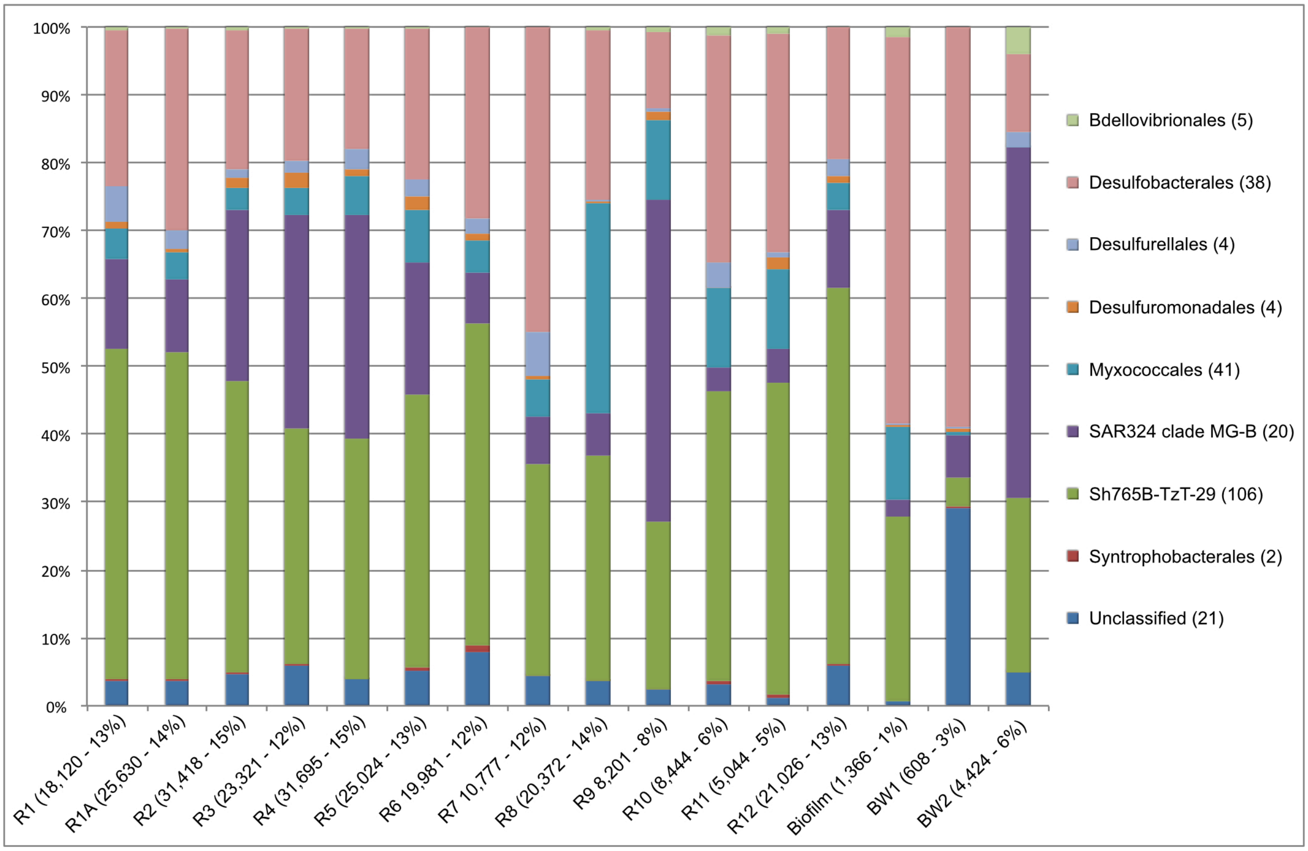

Figure S9: Breakdown of Deltaproteobacteria by order. In parentheses next to sample names on the x-axis are the total number of reads for each sample. In parentheses next to each taxon is the number of OTUs (97% similarity or greater) recovered in that taxon.
